# A Tc1‐ and Th1‐T‐lymphocyte‐rich tumor microenvironment is a hallmark of MSI colorectal cancer

**DOI:** 10.1002/path.6415

**Published:** 2025-04-03

**Authors:** Zhihao Huang, Tim Mandelkow, Nicolaus F Debatin, Magalie C J Lurati, Julia Ebner, Jonas B Raedler, Elena Bady, Jan H Müller, Ronald Simon, Eik Vettorazzi, Anne Menz, Katharina Möller, Natalia Gorbokon, Guido Sauter, Maximilian Lennartz, Andreas M Luebke, Doris Höflmayer, Till Krech, Patrick Lebok, Christoph Fraune, Andrea Hinsch, Frank Jacobsen, Andreas H Marx, Stefan Steurer, Sarah Minner, David Dum, Sören Weidemann, Christian Bernreuther, Till S Clauditz, Eike Burandt, Niclas C Blessin

**Affiliations:** ^1^ Institute of Pathology University Medical Center Hamburg‐Eppendorf Hamburg Germany; ^2^ College of Arts and Sciences Boston University Boston MA USA; ^3^ Department of Medical Biometry and Epidemiology University Medical Center Hamburg‐Eppendorf Hamburg Germany; ^4^ Institute of Pathology Clinical Center Osnabrück Osnabrück Germany; ^5^ Institute of Pathology Klinikum Fürth Fürth Germany; ^6^ Institute of Pathology, Arnold‐Heller‐Straße 3 University Medical Center Schleswig‐Holstein Kiel Germany

**Keywords:** colorectal cancer, microsatellite instability, T‐cell subpopulations, immune checkpoints, artificial intelligence, spatial analysis

## Abstract

Microsatellite instability is a strong predictor of response to immune checkpoint therapy and patient outcome in colorectal cancer. Although enrichment of distinct T‐cell subpopulations has been determined to impact the response to immune checkpoint therapy and patient outcome, little is known about the underlying changes in the composition of the immune tumor microenvironment. To assess the density, composition, degree of functional marker expression, and spatial interplay of T‐cell subpopulations, 79 microsatellite instable (MSI) and 1,045 microsatellite stable (MSS) colorectal cancers were analyzed. A tissue microarray and large sections were stained with 19 antibodies directed against T cells, antigen‐presenting cells, functional markers, and structural proteins using our BLEACH&STAIN multiplex‐fluorescence immunohistochemistry approach. A deep learning‐based framework comprising >20 different convolutional neuronal networks was developed for image analysis. The composition of Type 1 (T‐bet^+^), Type 2 (GATA3^+^), Type 17 (RORγT^+^), NKT‐like (CD56^+^), regulatory (FOXP3^+^), follicular (BCL6^+^), and cytotoxic (CD3^+^CD8^+^) or helper (CD3^+^CD4^+^) T cells showed marked differences between MSI and MSS patients. For instance, the fraction of Tc1 and Th1 was significantly higher (*p* < 0.001 each), while the fraction of Tregs, Th2, and Th17 T cells was significantly lower (*p* < 0.05) in MSI compared to MSS patients. The degree of TIM3, CTLA‐4, and PD‐1 expression on most T‐cell subpopulations was significantly higher in MSI compared to MSS patients (*p* < 0.05 each). Spatial analysis revealed increased interactions between Th1, Tc1, and dendritic cells in MSI patients, while in MSS patients the strongest interactions were found between Tregs, Th17, Th2, and dendritic cells. The additional analysis of 12 large sections revealed a divergent immune composition at the invasive margin. In summary, this study identified a higher fraction of Tc1 and Th1 T cells accompanied by a paucity of regulatory T‐cell, Th17, and Th2 T‐cell subpopulations, along with a distinct interaction profile, as a hallmark of MSI compared to MSS colorectal cancers. © 2025 The Author(s). *The Journal of Pathology* published by John Wiley & Sons Ltd on behalf of The Pathological Society of Great Britain and Ireland.

## Introduction

Colorectal cancer is the tumor entity with the highest degree of microsatellite instability ranging from 12% to 20% of cases [1, 2]. Microsatellite instable (MSI) colorectal cancers are well known for their strong link to favorable patient outcome [3], increased response to immune checkpoint inhibitors [4], high mutational burden [5], poor differentiation [6], and an inflamed immune phenotype [7]. Moreover, recent evidence suggests that the interdependencies between cytotoxic T cells and subpopulations of helper T cells, such as Th1, Th2, Th17, and Tregs highly impact the efficacy of anti‐cancer immunity depending on the microsatellite status [7, 8]. However, the alterations of the T‐cell composition and underlying changes in the immune tumor microenvironment (TME) that lead to such favorable patient outcomes are still unknown.

Microsatellite instability in colorectal cancer is associated with increased infiltration of CD8^+^ cytotoxic T cells, particularly Type 1 cytotoxic T cells (Tc1), leading to enhanced tumor cell destruction [8, 9]. Functional analysis has shown that type 1 cytotoxic T cells (Tc1) represent the main component of the terminal end route of anticancer immunity by producing high levels of cytolytic cytokines such as perforin, INF‐y, and granzyme B, thereby initiating direct tumor cell killing [8, 10], while other cytotoxic T‐cell subsets such as type 2 (Tc2) [8, 11] or regulatory cytotoxic T cells (Tcreg) [12] contribute less effectively to tumor cell destruction. Furthermore, it has been shown that type 1 T‐helper cells (Th1) can enhance Tc1 effector function by targeted delivery of cytokines via acquired pMHC I complexes [13]. In addition, Th1 is also well known for robust secretion of IFN‐γ and chemokines, facilitating the priming and proliferation of CD8^+^ T‐cell subpopulations [14, 15]. The role of Th2 and Th17 in the TME of colorectal cancer is more contradictory. Th2 has been found to promote tumor growth in colorectal cancer [16] but also shows antitumor activity due to its cytokine secretion profile [17]. Although a high fraction of Th17 was linked to tumor progression due to the promotion of intestinal tumorigenesis [18, 19] and angiogenesis by IL‐17 expression [20], anticancer functions such as promoting localization of highly cytotoxic CD8^+^ T cells to tumor tissues have also been described [21]. In addition, regulatory T cells (Tregs) appear to mitigate inflammatory damage and may suppress anticancer immune responses during different stages of carcinogenesis in colorectal cancer [22]. In view of the topographical location, degree of immune checkpoint expression, and proliferative state of these common nine T‐cell subpopulations, more than 40 functional and spatial T‐cell subpopulations can be identified in the immune TME in colorectal cancer.

To investigate the differences in composition, degree of functional marker expression, and spatial interplay of 45 T‐cell subpopulations between MSI and MSS colorectal cancers, an artificial intelligence‐based analysis of 19 marker BLEACH&STAIN multiplex fluorescence immunohistochemistry (mfIHC) was analyzed across 1,124 colorectal cancers.

## Materials and methods

### Patients and tissues

The study included a total of 1,309 colorectal cancers in a tissue microarray (TMA) format with tissue spots of diameter 0.6 mm (*n* = 1,297) and a set of large sections (*n* = 12) from patients operated upon between 2004 and 2019 at the University Medical Center Hamburg‐Eppendorf and the Institute of Pathology in the Klinikum Fürth, Fürth, Germany, with available data on the expression of MSH2, MSH6, MLH1, and PMS2 leading to the identification of 1,209 patients with a microsatellite stable (MSS) phenotype and 100 with a MSI phenotype. This cohort was not treated with immune checkpoint inhibitors. Detailed histopathologic data, including pathologic tumor stage (pT), pathologic lymph node status (pN), V stage, L stage, *RAS* mutation, or *HER2* amplification, were available from up to 1,286 tumors. Among 615 patients with sex and age data, 351 were male with an average age of 70, and 264 were female with an average age of 74. All samples came from the archives of the Institute of Pathology at the University Hospital of Hamburg (Hamburg, Germany) and Institute of Pathology at the Klinikum Fürth, Fürth, Germany. The use of archived remnants of diagnostic tissues for manufacturing of TMAs and their analysis for research purposes, as well as patient data analysis, had been approved by local laws (HmbKHG, x12) and by the local ethics committee (Ethics Commission Hamburg, WF‐049/09 25 January 2010). All work was carried out in compliance with the Declaration of Helsinki. Patient characteristics are described in supplementary material, Table S1.

### BLEACH&STAIN mfIHC

**Figure 1:**
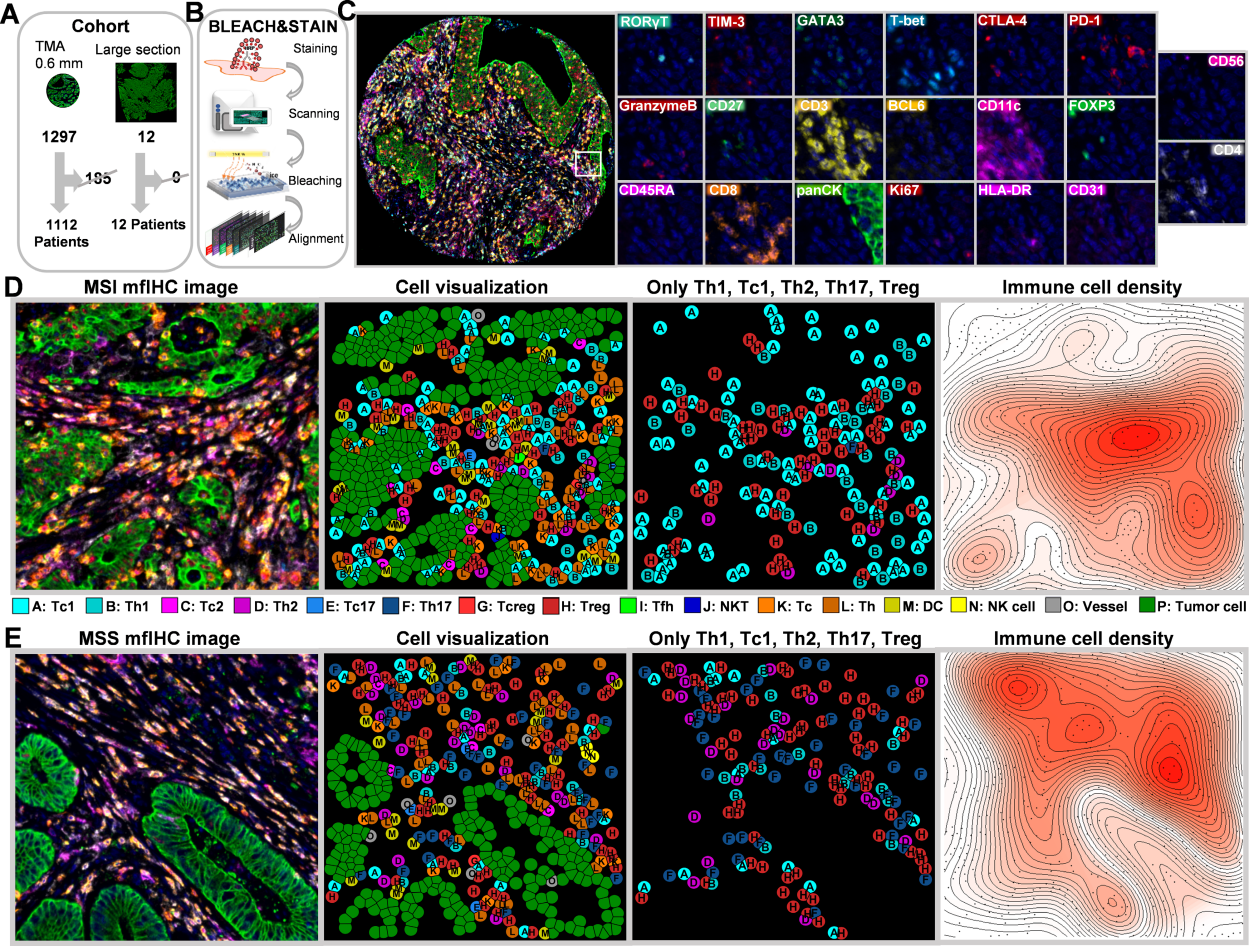
Patient cohort and BLEACH&STAIN framework. (A) 1,112 (85.7%) of 1,297 human colorectal cancer tissue samples in the 0.6 mm (tissue core diameter) TMA format and 12 (100%) of 12 patients in a large‐section format were analyzed in the study. For detailed patient characteristics see supplementary material, Figure S1 and Table S1. (B and C) The previously described BLEACH&STAIN multiplex fluorescence immunohistochemistry technology facilitates high‐throughput analysis of 20 antibodies that were stained in five sequential staining cycles (see supplementary material, Table S2). (D and E) Representative images of (D) a MSI and (E) a MSS T‐cell immune TME and its visualization through artificial intelligence‐based image analysis. The multiplex image for MSI shown here is the same as that used in Figure 3F.

Our recently developed BLEACH&STAIN [23] mfIHC approach that enables the analysis of 19 biomarkers and the spatial location on single cells in paraffin‐embedded and formalin‐fixed tissue using five sequential staining and imaging rounds of four biomarkers at a time and a bleaching step between every cycle was used in this study (Figure 1). The stained antibody panel of this study included CD3 (Polyclonal), CD8 (C8/144B), CD4 (MSVA‐004R), FOXP3 (206D), T‐bet (EP263), GATA3 (MSVA‐550R), RORγT(6F3.1), BCL6 (PG‐B6p), CD27 (MSVA‐027M), CD56 (MSVA‐056R), CD11c (EP1347Y), TIM‐3 (MSVA‐366R), PD‐1 (EPR4877(2)), CTLA‐4 (MSVA‐152R), panCK (MSVA‐000R), Ki67 (MSVA‐267R), CD31 (MSVA‐031M), GranzymeB (11F1), and HLA‐DR (MSVA‐470R). Details of the antibodies, antibody dilutions, antibody retrieval procedures, and OPAL dyes are given in supplementary material, Table S2, and details for BLEACH&STAIN mfIHC staining have been described in detail in the supplementary materials and methods, and elsewhere [24, 25, 26].

### Deep learning‐based framework for automated 19‐plex BLEACH&STAIN mfIHC image analysis

Image analysis was performed using the previously trained [23, 24] deep learning‐based (U‐Net) framework for cell detection, cell segmentation, intensity measurement of the used fluorophores (range 0–255, i.e. a continuous numerical value indicating the fluorescence signal strength), processing of intensity values, cell distance, and cell‐to‐cell interaction analysis using Python version 3.8 (RRID:SCR_008394) [27], R version 3.6.1 (The R Foundation) [28], and the Visiopharm software package (Hoersholm, Denmark). Marker positivity (i.e. CD3, CD8, CD4, FOXP3, T‐bet, GATA3, RORγT, BCL6, CD27, CD56, CD11c, TIM‐3, PD‐1, CTLA‐4, panCK, Ki67, CD31, GranzymeB, HLA‐DR, CD45RA) was evaluated by a deep‐learning (U‐Net) system for every marker individually that classified the marker as either positive or negative based on multiple features that have been identified by the convolutional neural network within the training set (e.g. the intensity level, distribution of the marker intensity across the cell, and cell shape). The novel deep‐learning systems (U‐Net) for marker positivity has been trained on 250 tissue samples using the deep‐learning frameworks Keras and Tensorflow [RRID: SCR_016345, in Python version 3.87 (RRID:SCR_008394)] and the Visiopharm software package. Marker‐positive cells were classified into 45 (functional) T‐cell subpopulations according to unsupervised X‐shift clustering (supplementary material, Figure S1A,B), the expression profile of well‐characterized subpopulations, and their functional state (supplementary material, Figure S1C). A pathologist reviewed every tissue sample to exclude tissue spots that were lacking cancer cells. The invasive margin in large sections was defined as an area expanding 360 μm into the stroma and 360 μm into the tumor measured from the stroma‐tumor borderline [29] and was selected manually by a pathologist. The detailed image analysis has been described in detail in the supplementary materials and methods, and elsewhere [24, 25, 26].

### Spatial analysis

A cell‐to‐cell contact was defined as the distance of eight or fewer micrometers between the center points of two cells, as described in our previous study [24, 25, 26]. R version 3.6.1 (The R Foundation) [28] was used to calculate the nearest distance between every cell subpopulation. The proportion of normalized interactions, ‘(p)‐normalized interactions,’ was calculated by dividing the number of cell‐to‐cell contacts of two interacting immune cell subpopulations by the overall number of cell‐to‐cell contacts of all analyzed immune cells. The ‘normalized interactions’ were calculated by dividing the number of cell‐to‐cell contacts of two interacting immune cell subpopulations by the number of cells in these two immune cell subpopulations. The number of random relative interactions (noise) was calculated by measuring the number of all cell‐to‐cell contacts per area and used to estimate whether the relative cell‐to‐cell counts of the immune cell subpopulations were significantly enriched – compared to the random ‘background’ interactions – or just by chance. Dense accumulations of T‐cells, accompanied by an increase in cell‐to‐cell contacts, were identified as T‐cell nests (supplementary material, Figure S2A–E). The DBSCAN algorithm (R dbscan package, R opticskxi package) was used to identify T‐cell accumulation. MinPts was set as 27 to define the minimum number of cells in a T‐cell nest (supplementary material, Figure S2A,B). Eps was identified by running the DBSCAN algorithm in different Eps values on manually selected T‐cell accumulation (supplementary material, Figure S2C,D). The crossing point between the sensitivity and specificity curves showing the optimal Eps that was found was 40 (supplementary material, Figure S2C).

### Statistical analyses

Statistical calculations were performed using R version 3.6.1 (RRID:SCR_001905, The R Foundation) [28, 30] and JMP Pro 17 software package (RRID:SCR_022199) [31]. The clinical and histopathological parameters of the patients were reported as counts and percentages for categorical data and compared using Pearson's 𝜒^2^ tests. All continuous variables in different groups were compared using ANOVA (JMP Pro 17 software package or R stats package). Immune parameters between groups were compared using a *t*‐test. In the case of large‐section analysis, the regions were randomly separated into 48 regions for comparison and heterogeneity analysis. Unsupervised X‐shift clustering [32] was applied to differentiate patient subgroups based on their marker expression pattern (VorteX software). The clustering via X‐shift was performed using the number of nearest neighbors (K) method, and the optimal value for K (20) was determined via elbow points and resulted in 242 clusters. All ratios with a denominator of zero were treated as having no value and excluded from the analysis. All *p* values were two‐sided, and *p* < 0.05 was considered significant.

## Results

### Technical aspects

A total of 1,112 (85.7%) of 1,297 human colorectal cancer tissue samples in the 0.6‐mm (tissue cores in diameter) TMA format were interpretable in this study (Figure 1). The remaining 185 tumor samples were excluded due to the complete loss of tissue spots or the lack of unequivocal cancer cells.

### Composition and density between MSI and MSS

**Figure 2:**
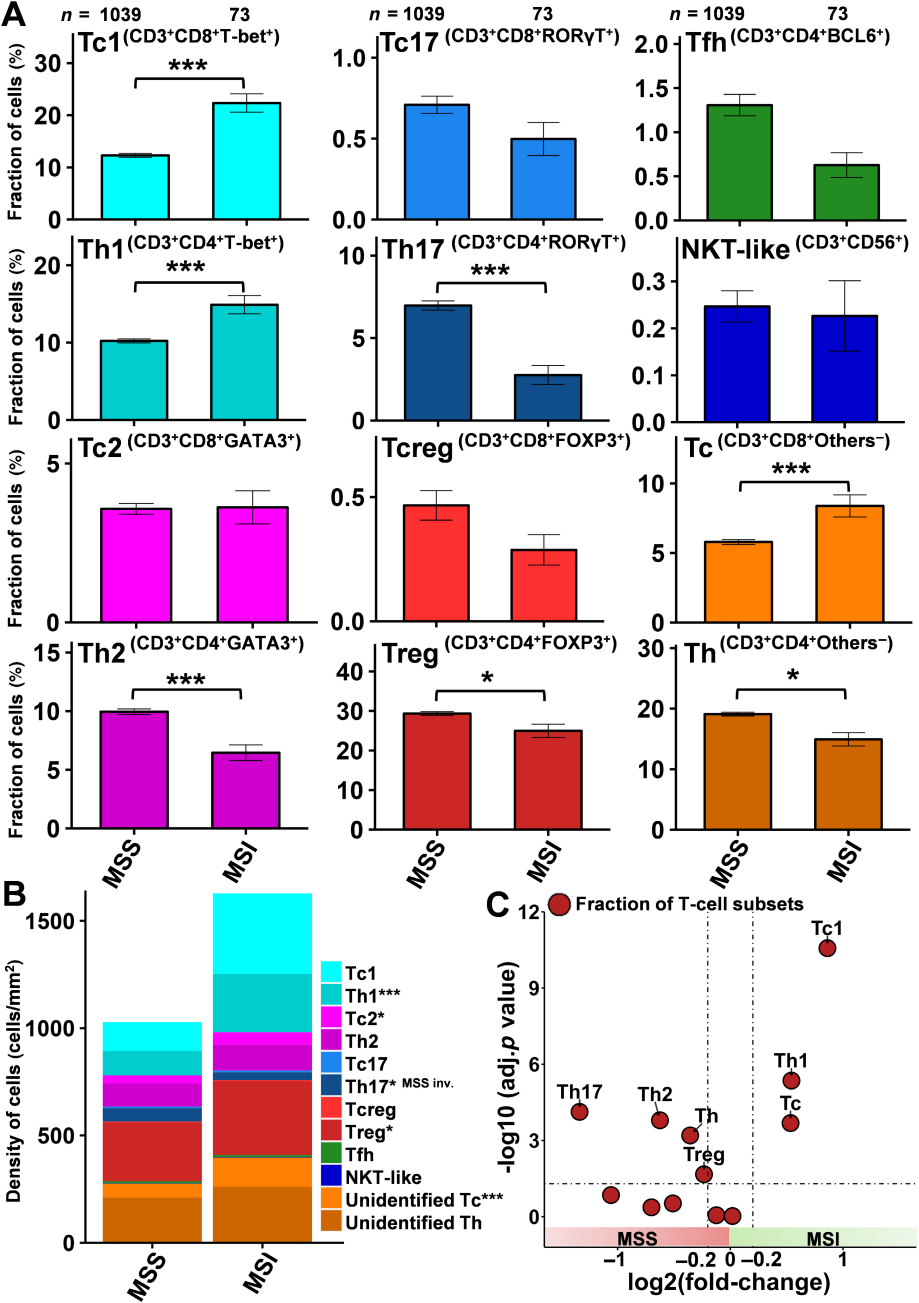
Fraction and density of T‐cell subsets between MSS and MSI colorectal cancers. (A) The proportions (%) of T‐cell subsets for MSS and MSI colorectal cancers. Error bars: SEM of each fraction. *p < 0.05, **p < 0.01, and ***p < 0.001. (B) The total cell density (cells/mm^2^) of T‐cell subpopulations is shown for MSS and MSI colorectal cancers. ***p < 0.001. (C) Volcano plot depicting log_2_ fold‐change on *x*‐axis and −log_10_ adjusted *p* values on *y*‐axis of fraction (%) of T‐cell subsets in MSI patients compared to MSS patients.

Although the abundance (i.e. density in cells/mm^2^) of most T‐cell subpopulations was significantly higher in 73 MSI (1,628 ± 1,687, MSI) compared to 1,039 MSS (1,028 ± 1,113, MSS) colorectal cancers (*p* < 0.05 each), the fraction of Type 1 (T‐bet^+^), Type 2 (GATA3^+^), Type 17 (RORγT^+^), regulatory (FOXP3^+^), and follicular (BCL6^+^) cytotoxic (CD3^+^CD8^+^) or helper (CD3^+^CD4^+^) T cells showed marked differences between MSI and MSS patients (Figure 2A,B and supplementary material, Figure S3). For instance, the fraction of Tc1 and Th1 as well as other cytotoxic T cells was significantly enriched in MSI patients (*p* < 0.001 each), while the fraction of Th2, Th17, Tregs, and other T‐helper cells (*p* < 0.05 each) was significantly higher in MSS colorectal cancers (Figure 2A,B). The strongest difference was seen between MSI and MSS colorectal cancers for the dramatically enriched fraction of Tc1 in MSI patients (Figure 2C). Three representative images for both MSI and MSS cores are shown in supplementary material, Figure S4. The correlation plot found only a very weak positive association between fraction of Tc1 with Th1 (*r* = 0.13) (supplementary material, Figure S5). In MSS cancers, a higher fraction of Th2 and Tc2 was associated with advanced pT stage (*p* = 0.007 and *p* < 0.001, respectively), and a higher fraction of Th17 and Tc17 was significantly linked to lower pT stage (*p* = 0.03 and *p* = 0.01, respectively, supplementary material, Table S3). In addition, a higher fraction of Th2 was associated with nodal metastasis (*p* = 0.02, supplementary material, Table S3). In the MSI subgroup, only a low fraction of Th1 and a high fraction of Tc2 were associated with nodal metastasis (*p* = 0.03 and *p* = 0.003, respectively, supplementary material, Table S4).

### Functional marker expression in MSI and MSS

**Figure 3:**
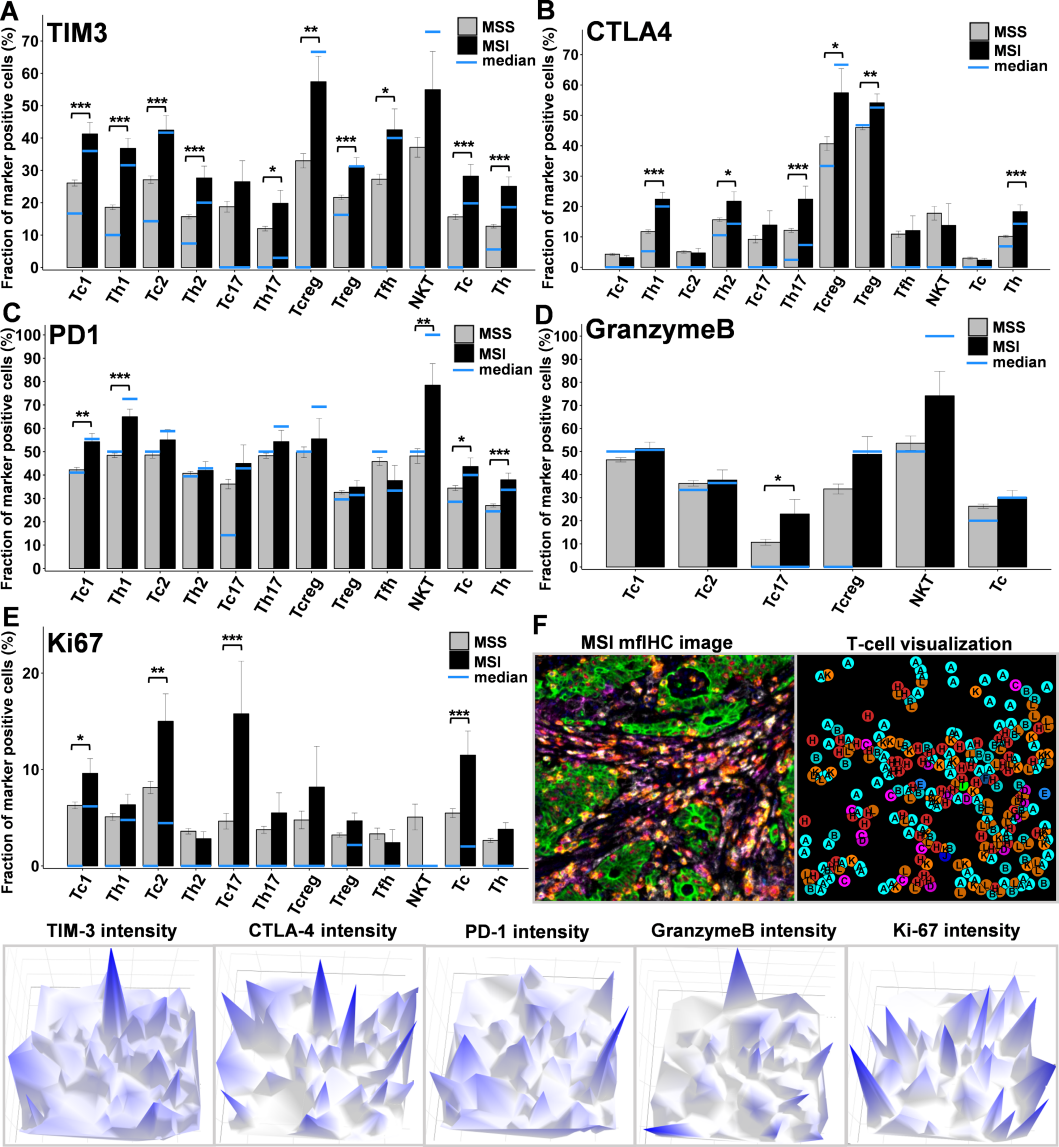
Functional markers compared between MSI and MSS. (A–E) Fraction (%) of marker‐positive cells is shown for each T‐cell subpopulation between patients with MSI (black) and MSS (gray) tumors. The blue segments inside or outside the bars indicate the median value of each fraction. Error bars: SEM of each fraction. *p < 0.05, **p < 0.01, and ***p < 0.001. (F) A representative image, its visualization, and three‐dimensional plots showing functional marker expression in TME of MSI patients. The multiplex image for MSI shown here is the same as used in Figure 1D.

The fraction of TIM3 expressing cells was significantly higher in MSI compared to MSS patients in almost all analyzed cytotoxic and helper T‐cell subpopulations (*p* < 0.05, Figure 3A). A significantly higher fraction of CTLA‐4 expression in MSI was found for Th1, Th17, and Tregs (*p* < 0.01, Figure 3B), while increased PD‐1 expression in MSI patients was seen in both Tc1 and Th1 cells (*p* < 0.01, Figure 3C). An increased fraction of Granzyme B positive cells was detected only for Tc17 (*p* < 0.05, Figure 3D). The proliferation rate was found to be increased for Tc1, Tc2, and Tc17 in MSI compared to MSS patients (*p* < 0.05 each, Figure 3E). The relative intensity of functional marker for a MSI representative image is shown in Figure 3F.

### Spatial interplay between MSI and MSS patients

**Figure 4:**
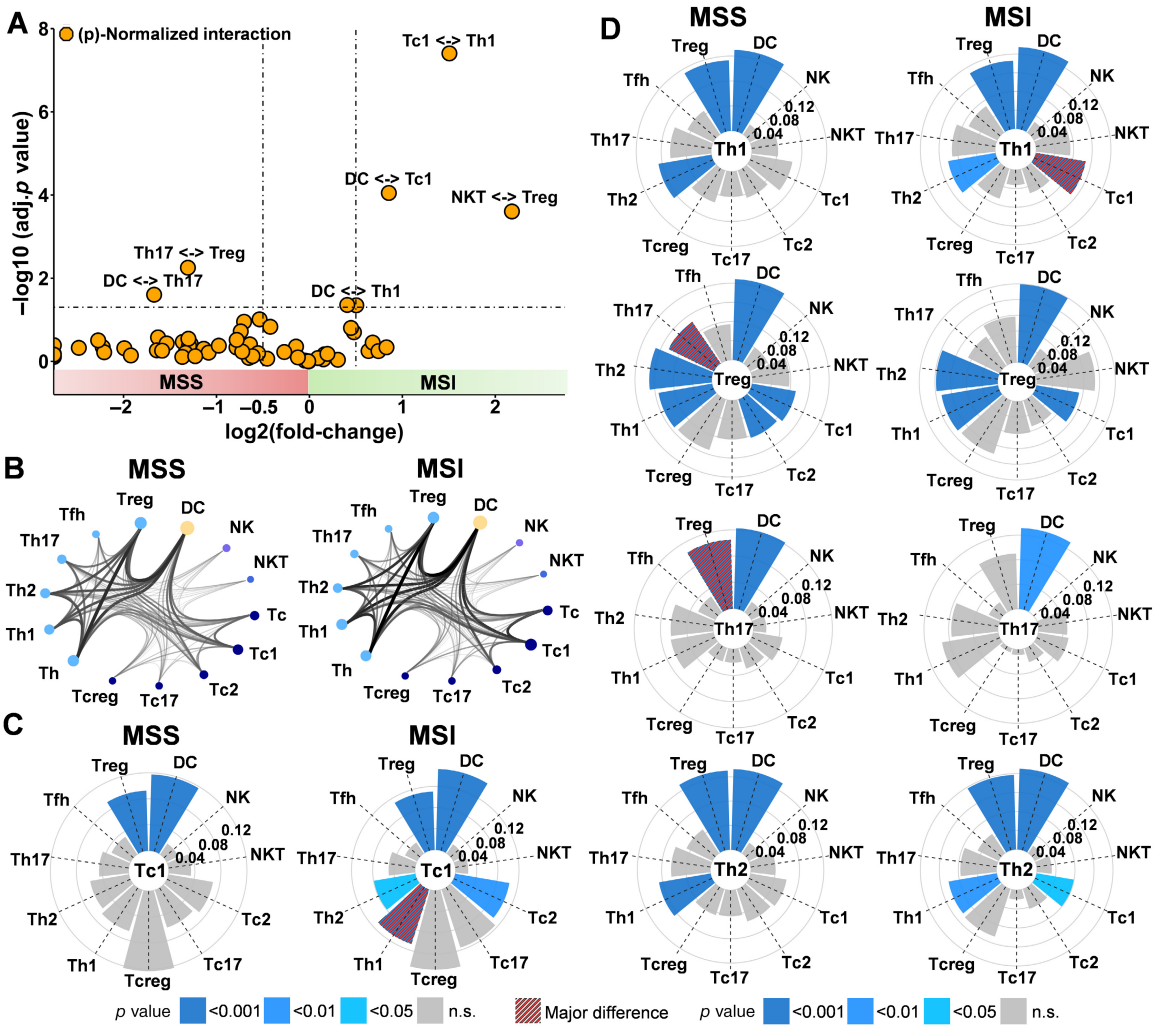
Cell‐to‐cell interactions compared between MSI and MSS. (A) Volcano plot depicting log_2_ fold‐change on *x*‐axis and −log_10_ adjusted *p* values on *y*‐axis of (*p*) normalized cell‐to‐cell interactions of T‐cell subsets, dendritic cells, and NK cells in MSI patients versus MSS patients. (B) Profile of normalized cell‐to‐cell interactions of T‐cell subsets, dendritic cells, and NK cells in MSI patients versus MSS patients. The thickness of the connections in the cord plots correlates with the number of relative cell interactions, and the size of the nodes indicates the number of cells per cell subpopulation. (C and D) Circular bar plots indicate the significance of normalized cell‐to‐cell interactions compared to the background noise (size, shades of blue and gray), and the significant differences between MSS and MSI are highlighted in red.

Comparison of relative cell‐to‐cell contacts [(p)‐normalized cell interactions] between MSI and MSS patients revealed a markedly increased interaction between Th1 and Tc1 as well as dendritic cells and Tc1 cells in MSI patients (*p* < 0.001, Figure 4A). The spatial orchestration in MSS patients was characterized by the strongest (p)‐normalized interactions between Th17 and Tregs as well as dendritic cells (*p* < 0.05, Figure 4A). Of note, a significant normalized interaction (i.e. significant interactions compared to the background of random interactions) between Tc1 and Th1 was exclusively seen in MSI patients but was absent in MSS patients (*p* < 0.001, Figure 4B–D). A significant normalized interaction between Tregs and Th17 was exclusively detected in MSS patients (*p* < 0.001, Figure 4B–D).

### MSI‐like immune phenotype in MSS patients

X‐shift clustering of 1,039 microsatellite stable colorectal cancer patients revealed that 87 (8.4%) MSS patients also paralleled the immune phenotype of microsatellite instability in colorectal cancer and were thus considered MSI‐like (supplementary material, Figure S6A,B). These MSI‐like colorectal cancers showed an enriched fraction of Tc1 and Th1 accompanied by a low fraction of Th2, Th17, and Tregs with the strongest difference in the fraction of Tc1 cells compared to the other MSS patients (each *p* < 0.001, supplementary material, Figure S6C,D). The expression level of TIM3, CTLA‐4, and PD‐1 was also significantly enriched in most T‐cell subpopulations in MSI‐like cancers compared to other MSS patients (supplementary material, Figures S6E and S7A–E). Spatial analysis revealed that the strongest interaction network was seen again for Tc1 and dendritic cells as well as for Th1 and Tc1 in MSI like patients, while the strongest relative interactions in other MSS patients were also seen between Tregs, Th17, Th2, dendritic cells, and other helper T‐cells (supplementary material, Figures S6F,G and S7F).

### Large‐section validation and CT versus IM

**Figure 5:**
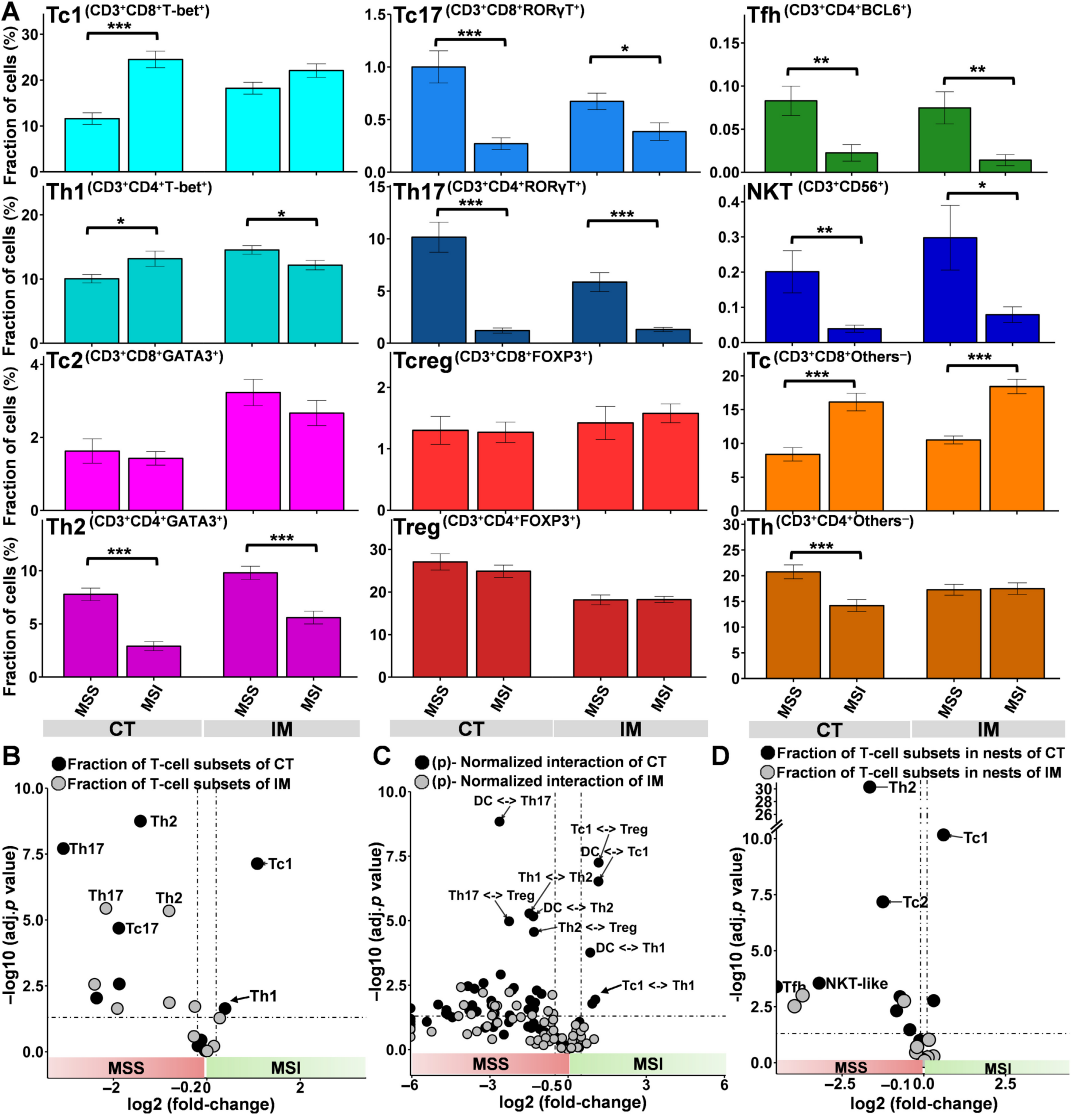
Large‐section validation and difference between MSI and MSS tumors in center (CT) versus invasive margin (IM). (A) The fraction (%) of T‐cell subsets is shown between MSS and MSI colorectal cancers in both the CT and at IM across 12 large sections. Error bars: SEM of each fraction. *p < 0.05, **p < 0.01, and ***p < 0.001. (B–D) Volcano plot depicting (B) log_2_ fold‐change on *x*‐axis and −log_10_ adjusted *p* values on *y*‐axis of T‐cell fraction, (C) (p)‐normalized cell‐to‐cell interactions, and (D) fraction of T‐cell subsets in T‐cell nests compared between MSI and MSS tumors.

Our additional analysis of 12 large sections of six MSI and six MSS colorectal cancers confirmed the characteristic immune phenotype of MSI colorectal cancers (Figure 5A,B and supplementary material, Figure S8) and the increased level of immune checkpoint expression in MSI patients in the center of the tumor (supplementary material, Figure S9). The comparison of (p)‐normalized interactions also confirmed dramatically increased interaction between Th1 and Tc1 (*p* < 0.05, Figure 5C) as well as dendritic cells and Tc1 cells (*p* < 0.001, Figure 5C) in MSI patients compared to MSS patients in the center of the tumor. However, the characteristic composition and interactions of T‐cell subpopulations found in the center of the tumor in both the TMA and the large‐section cohort were not found at the invasive margin (Figures 2 and 5A,B). Additional T‐cell nest analysis identified enrichment of the fraction of Tc1 (*p* < 0.001, Figure 5D) located in T‐cell nests of the center of the tumor but not at the invasive margin of MSI patients compared to MSS patients (Figure 5D).

## Discussion

Finding a significantly higher fraction of Tc1 and Th1 accompanied by an increased immune checkpoint expression in MSI than in MSS colorectal cancer has also been suggested by other studies using non‐immunohistochemistry‐based methods. A study on 598 colorectal cancers employing next‐generation sequencing also reported a higher gene expression of Th1 related signatures in MSI colorectal cancers [33]. Using RT‐qPCR in a cohort of 25 colorectal cancers, Llosa *et al* showed a higher expression of Type 1 cytotoxic and Type 1 helper T‐cell‐related genes in MSI compared to MSS colorectal cancers [34]. Utilizing single‐cell RNA‐sequencing analyses, others demonstrated a significantly increased Th1‐like cell signal in MSI patients in a cohort of 12 colorectal cancers [35]. A higher expression of Th1‐related signatures in MSI compared to MSS colorectal cancers was also observed in a dataset from The Cancer Genome Atlas from 270 colorectal adenocarcinomas [7]. In addition, these authors found a higher expression of Th2‐associated genes in MSI colorectal cancers, which is not supported by our results. There is also growing evidence for increased TIM3 [36], CTLA‐4 [34], and PD‐1 [7] expression in MSI compared to MSS colorectal cancers. The concordance with our findings using a variety of methods can be seen as an indirect validation of our multiplex fluorescence BLEACH&STAIN analysis framework.

The increased fraction of Tc1 cytotoxic T cells and Th1 helper T cells accompanied by a strong interaction network between Tc1, Th1, and dendritic cells in MSI colorectal cancers compared to MSS patients suggests a driving role in the enhanced anticancer immunity of a MSI TME. Thus, these findings correspond well to growing evidence that Th1 cells can acquire the ability to promote Tc1 cell survival, effector function, and tumor‐adjacent localization by targeted delivery of cytokine IL‐2 [13, 37]. Likewise, Tc1 cells were observed to promote the polarization of CD4^+^ T cells toward a Th1 phenotype, enhancing the Tc1/Th1‐mediated anticancer immune response [9]. These findings fit well with the fact that distinct dendritic cell subpopulations can present tumor antigens to promote polarization of CD8^+^ T cells into Type 1 cytotoxic T cells (Tc1) [38, 39]. In agreement with this, recent evidence has demonstrated that vaccine‐based rescue of dendritic cells can result in *in vitro* and *in vivo* cytotoxic T‐cell responses due to elevated Tc1 levels [40]. Given that Tc1 cells are known as a cytotoxic T‐cell subpopulation with superior cytotoxicity against tumor cells compared to other cytotoxic T‐cell subsets [8, 9, 10], the ‘terminal end route’ of anticancer immunity due to direct cell‐to‐cell contact‐based tumor cell elimination might be represented by a high fraction of Tc1 cells that are in direct contact with enhancing Th1 and dendritic cells.

Finding a reduced fraction of Th17, Th2, and regulatory T cells, along with the absence of a strong interaction network between these T cells in MSI colorectal cancers but finding an enriched fraction and strong interaction network between Th17, Th2, Tregs, and dendritic cells in MSS patients highlights a less conducive pro‐tumorigenic microenvironment in MSI colorectal cancers and a pro‐tumorigenic TME in MSS patients. Hence, there is evidence for a tumor‐promoting role of Th2 in colorectal cancers as a result of promoting epithelial to mesenchymal transition [16], tumor cell proliferation, invasion [41], and initiation as well as progression of colorectal cancer [42]. In addition, some studies have shown that distinct dendritic cell subpopulations can promote Th2 differentiation and, thus, tumor progression [43, 44], which fits very well with the strong interaction between DCs and Th2 exclusively found in MSS patients and its absence in the TME of MSI patients. However, the role of Th17 in colorectal cancer is controversial. Experimental models have demonstrated that Th17 cells contribute to intestinal tumorigenesis [18, 19] and promote angiogenesis [20]. In contrast, it has also been shown that Th17 can recruit highly cytotoxic CD8^+^ T cells in the intraepithelial tumor compartment [21].

The identification of increased levels of immune checkpoint expression, particularly of TIM3, CLTA‐4, and PD‐1, in MSI colorectal cancers that are well known for a high pre‐existing antitumor immunity underlines the concept that high levels of immune checkpoint expression do not hinder anticancer immunity in an inflamed TME. These findings are in line with data from our recent study in muscle‐invasive urothelial carcinomas providing an example where a high expression of TIM3, PD‐1, and CTLA‐4 on immune cell subpopulations was linked to a favorable outcome in an inflamed immune phenotype [26]. Others have also shown in several different entities that high levels of immune checkpoint expression do not necessarily imply a terminally exhausted immune environment [45]. Thus, these findings underline the concept that a high degree of immune cell infiltration (i.e. a high degree of anticancer immunity) is accompanied by a physiologically necessary upregulation of immune checkpoint receptors to prevent an excessive unregulated ‘overinflammation’ or, eventually, autoimmune reaction [46].

Finding the characteristic enrichment of Tc1 and Th1 in MSI patients compared to MSS patients in the center of the tumor but not at the invasive margin underlines the concept that the immune TME (iTME) in proximity to the cancer cells – thus in the center of the tumor – relies on different immune regulations compared to the iTME at the invasive margin. It is well known that the abundance of immune cells, particularly T‐cell subpopulations is on average two to three times higher at the invasive margin compared to the center of the tumor [47, 48]. Accordingly, immune suppressive T‐cell subpopulations, such as regulatory T cells and tumor‐associated macrophages, were also described as being enriched at the invasive margin compared to the center of the tumor [48, 49]. Furthermore, the accumulation of (PD‐L1^+^) myeloid cells and increased interactions with PD‐1^+^ T cells at the invasive margin was found to contribute to the immune suppressing activity and drive tumor immune evasion [49, 50]. Moreover, the center of the tumor is known to have a hypoxic TME, while the invasive margin is known for an oxygen‐rich TME that regulates T‐cell functionality [49]. Therefore, the invasive margin and center of the tumor can be interpreted as two completely different compartments of the immune TME, and the data from this study suggest assessment of T‐cell subpopulations in the center of the tumor of colorectal cancers.

This study examined a wide range of cell types and immune parameters. However, a key limitation is the relatively small number of patients, particularly the imbalance within the cohort of 1,112 colorectal cancer cases, which included only 73 MSI tumors compared to 1,039 MSS tumors. This disparity, combined with the large number of statistical analyses performed, increases the risk of false‐positive statistical findings. There was, however, a subset of 8% of colorectal cancer patients with a MSI‐like immune phenotype among the MSS colorectal cancers that was characterized by elevated levels of Type 1 cytotoxic T cells (Tc1) and Type 1 helper T cells (Th1) accompanied by reduced levels of Th17, Th2, and regulatory T cells and a strong interaction network. This fits well with data from several other studies describing a MSI‐like phenotype in subsets of 14–270 MSS cancers [7, 34, 51]. It will be important to determine whether these MSI‐like cancers represent the 0.8%–12% of MSS colorectal cancers that have been reported to positively respond to immune checkpoint blockade [52, 53, 54]. In line with this notion, Llosa *et al* recently identified a cluster of about 10% of MSS patients characterized by elevated PD1^+^Tc1 cells, elevated PD‐L1 expression, and diminished IL‐17^+^ T cells, which showed significant response to anti‐PD‐1 monotherapy [51].

In conclusion, this study has identified that a higher fraction of Type 1 cytotoxic T cells (Tc1) and Type 1 helper T cells (Th1) with a strong and distinct spatial interplay accompanied by a paucity of Th17, Th2, and regulatory T cells is a characteristic feature of MSI colorectal cancers, especially in comparison to MSS patients that showed an adverse spatial interaction profile.

## Author contributions statement

NCB and GS conceived and designed the study. ZH, TM, NFD, EB, JHM, RS, AM, KM, NG, ML, AML, DH, TK, PL, CF, AH, FJ, AM and SS acquired the data. ZH and JE performed the experiments. ZH, TM, JBR, EV, DD and NCB analyzed and interpreted the data. GS, EB, SM, SW, CB, TSC and NCB critically revised the manuscript for important intellectual content. ZH, TM, JBR and NCB performed the statistical analyses. GS and NCB supervised the study.

## Supporting information


Supplementary materials and methods
**Figure S1.** Identification of cell subpopulations
**Figure S2.** Nest detection
**Figure S3.** Fraction of T‐cell subsets between MSS and MSI colorectal cancers
**Figure S4.** Representative images for MSI and MSS cores
**Figure S5.** Association between fraction of Tc1 with Th1
**Figure S6.** MSI‐like immune phenotype in a subcohort of MSS patients
**Figure S7.** Functional markers and cell‐to‐cell interactions between MSI‐like and MSS
**Figure S8.** Difference of fraction of T‐cell subsets between MSI and MSS patients in CT versus IM
**Figure S9.** Functional markers between MSI and MSS in center of tumor of large sections
**Table S1.** Patient characteristics shown for 0.6‐mm TMA cores analyzed
**Table S2.** List of used antibodies, antigen retrieval (AR), dilutions, and Opal dyes for mfIHC
**Table S3.** Association of pT, pN with fractions of T‐cell subsets in MSS patients
**Table S4.** Association of pT, pN with fractions of T‐cell subsets in MSI patients

## Data Availability

Data are available for *bona fide* researchers who request it from the authors.
