# Supplementary material for: A Tc1‐ and Th1‐T‐lymphocyte‐rich tumor microenvironment is a hallmark of MSI colorectal cancer
Source: J Pathol. 2025 Apr 3;266(2):192–203. doi: 10.1002/path.6415 (PMC12056287; doi:10.1002/path.6415)
Supplement: Supplementary file 1 — Supplementary materials and methods Figure S1. Identification of cell subpopulations Figure S2. Nest detection Figure S3. Fraction of T‐cell subsets between MSS and MSI colorectal cancers Figure S4. Representative images for MSI and MSS cores Figure S5. Association between fraction of Tc1 with Th1 Figure S6. MSI‐like immune phenotype in a subcohort of MSS patients Figure S7. Functional markers and cell‐to‐cell interactions between MSI‐like and MSS Figure S8. Difference of fraction of T‐cell subsets between MSI and MSS patients in CT versus IM Figure S9. Functional markers between MSI and MSS in center of tumor of large sections Table S1. Patient characteristics shown for 0.6‐mm TMA cores analyzed Table S2. List of used antibodies, antigen retrieval (AR), dilutions, and Opal dyes for mfIHC Table S3. Association of pT, pN with fractions of T‐cell subsets in MSS patients Table S4. Association of pT, pN with fractions of T‐cell subsets in MSI patients [file PATH-266-192-s001.docx]

**A Tc1- and Th1-T-lymphocyte-rich tumor microenvironment is a hallmark of MSI colorectal cancer**

Z Huang, T Mandelkow *et al. J Pathol* <https://doi.org/10.1002/path.6415>

**Supplementary materials and methods**

**Supplementary Figures S1–S9**

**Supplementary Tables S1–S4**

**Supplementary materials and methods**

## BLEACH&STAIN multiplex fluorescence immunohistochemistry (mfIHC).

Several primary antibody clones for a single target were compared in preliminary experiments, as described earlier [55], and the optimal dilution and pH for every antibody clone, composed in the 19+1 marker BLEACH&STAIN panel, were evaluated on a test TMA using conventional brightfield immunohistochemistry. Freshly cut 4-µm consecutive tissue sections were used on X-tra^®^ glass slides (Catalogue No. 3800204AE, Leica), with a positively charged surface, for mfIHC staining. The 19+1 marker BLEACH&STAIN mfIHC was conducted, as described previously [23], in five sequential staining and imaging rounds of four biomarkers at a time and a bleaching step between every cycle. Slides were initially boiled in an autoclave (30 min at 100–120 °C) for antigen retrieval. The staining of a single biomarker was performed largely according to the manufacturer’s instructions (AKOYA). In brief, one cycle of antibody staining included peroxidase blocking, application of the primary antibody, detection with a secondary HRP-conjugated antibody, fluorescence dye detection, and removal of the bound antibodies by microwave treatment (5 min at 100 °C and 5 min at a mean temperature of 93 °C). This cycle was repeated three times for the remaining antibodies. Slides were subsequently counterstained with DAPI and mounted in an antifade solution. The fluorescence dyes OPAL 520 (Catalogue No. FP1487001KT AKOYA Biosciences, Menlo Park, CA, USA), OPAL 570 (Catalogue No. FP1488001KT, AKOYA Biosciences), OPAL 620 (Catalogue No. FP1495001KT, AKOYA Biosciences), and OPAL 690 (Catalogue No. FP1497001KT, AKOYA Biosciences) were used for visualization of antibody binding. Details on antibody dilutions, antibody retrieval procedures, and OPAL dyes are given in supplementary material, Table S2. Digital images of mfIHC slides were acquired using a Leica Aperio VERSA 8 automated epifluorescence microscope and AKOYA’s PhenoImager^TM^ HT slide scanner. The bleaching step between every sequential 4+1 marker staining included photobleaching (1,600-W metal-halide lamp) combined with slide incubation in 3% hydrogen peroxide for chemical inactivation of fluorochromes and cooling (4 °C) of the slides during the bleaching process. The bleaching process was stopped as soon as the fluorescence signal of all fluorochromes had vanished (exposure time >600 ms). Finally, the four sequential digital images were aligned and thus merged into a single 19+1 mfIHC image using custom software written in the Python programming language [27] (Figure 1).

## Deep learning-based framework for 19-plex BLEACH&STAIN mfIHC image analysis.

ThIimage analysis was performed according to the following steps using Python version 3.8 (RRID:SCR_008394)[27], R version 3.6.1 (The R foundation, R Project for Statistical Computing (RRID:SCR_001905)[28], and the Visiopharm software package (VIS Image Analysis Software (RRID:SCR_021711), Hoersholm, Denmark):

(1) The TMAs were segmented and a previously trained [23] DeepLabv3^+^ was used to quantify the area of every individual TMA spot and large-section regions (i.e. each patient).

(2) To identify individual cells (i.e. cell segmentation), a previously trained deep learning-based (U-Net) framework for segmentation of cell nuclei and the adjacent cytoplasm was used [24]. Thus, the intensity of the used fluorophores (range 0–255, i.e. a continuous numerical value indicating the fluorescence signal strength) in the nuclear and cytoplasmic cell compartment, as well as the localization of every cell, were documented in per-cell data.

(3) Marker positivity (i.e. CD3, CD8, CD4, FOXP3, T-bet, GATA3, RORγT, BCL6, CD27, CD56, CD11c, TIM-3, PD-1, CTLA-4, panCK, Ki67, CD31, GranzymeB, HLA-DR) was evaluated by a deep-learning (U-Net) system for every marker individually that classified the marker as either positive or negative based on multiple features that had been identified by the convolutional neural network within the training set (e.g. intensity level, distribution of marker intensity across cells, and cell shape). The novel deep-learning systems (U-Net) for marker positivity was trained on 250 tissue samples using the deep-learning frameworks Keras and Tensorflow (RRID: SCR_016345, in Python version 3.87) and the Visiopharm software package.

(4) For the identification and definition of immune cell subpopulations, unsupervised X-shift clustering was applied and revealed 242 subpopulations (supplementary material, Figure S1A,B). Within these subpopulations, the well-characterized expression profile of Tc1 (CD3^+^CD8^+^T-bet^+^), Th1 (CD3^+^CD4^+^T-bet^+^), Tc2 (CD3^+^CD8^+^GATA3^+^), Th2 (CD3^+^CD4^+^GATA3^+^), Tc17 (CD3^+^CD8^+^ RORγT^+^), Th17 (CD3^+^CD4^+^ RORγT^+^), Tcreg (CD3^+^CD8^+^FOXP3^+^), Treg (CD3^+^CD4^+^FOXP3^+^), Tfh (CD3^+^CD4^+^BCL6^+^), NKT-like (CD3^+^CD56^+^), dendritic cells (CD11c^+^), and tumor cells (panCK^+^) was identified. These 12 main subsets were further subclassified in 54 immune and tumor cell subpopulations according to their functional state (proliferation, cytotoxicity, immune checkpoint expression; see supplementary material, Figure S1C).

(5) The per-cell data derived from the image analysis framework represent the input data to assess spatial interactions (cell-to-cell contact), cell distances (µm), T-cell densities (cells/mm^2^), T-cell composition (%), fraction of functional marker-positive cells (%), and T-cell accumulation.

**Supplementary Figures S1–S9**


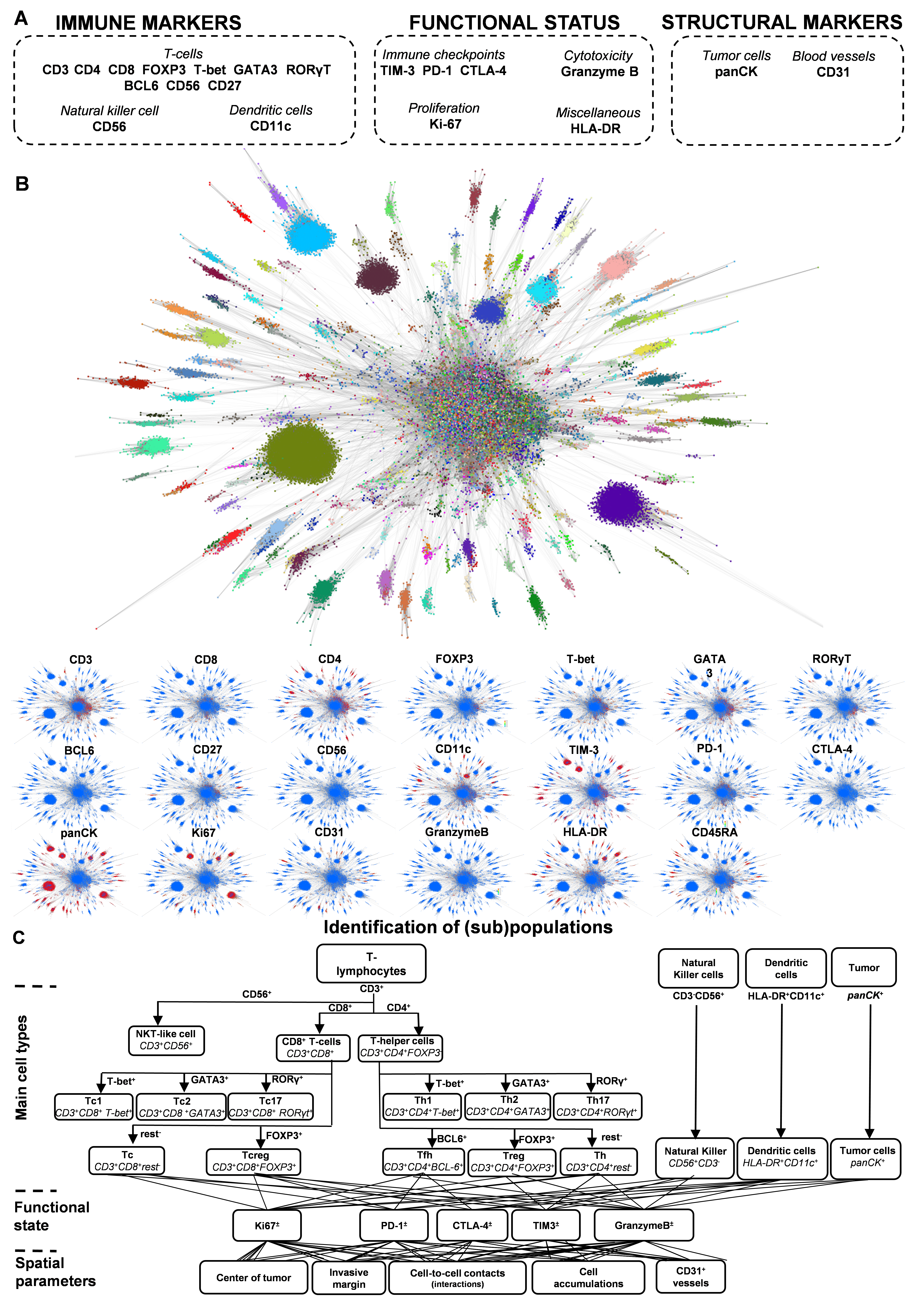


**Figure S1. Identification of cell subpopulations.**

(A) Organizational grouping of used 19 markers into three groups.

(B) Unsupervised X-shift clustering identified 242 immune cell subpopulations.

(C) Expression profile used to identify 54 immune and tumor cell subpopulations according to cell type and its functional state (see also Section S1B).

##
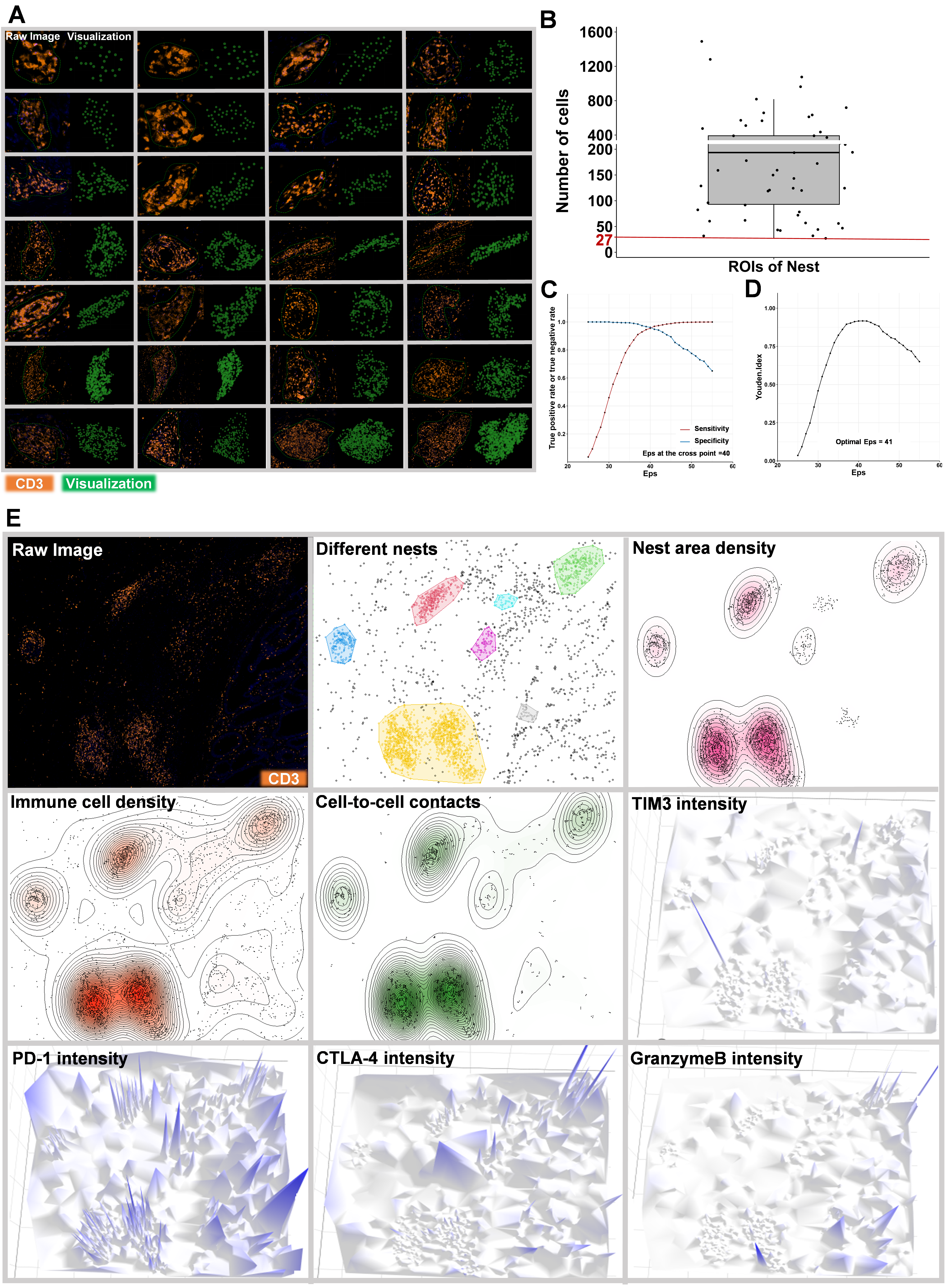


## Figure S2. Nest detection.

## (A) Representative images and T-cell visualization showing manually selected regions of the nest.

## (B) Box plot showing number of cells in 60 different nest regions. The red line indicates that the minimum number of cells is 27.

## (C) Specificity and sensitivity curves showing accuracy of nest detection based on range of Eps from 25 to 55. The Eps at the cross point is 40.

## (D) Youden's index curve showing accuracy of nest detection based on a range of Eps from 25 to 55. The Eps at optimal Youden's index is 41.

## (E) Representative images showing nests detected by DBSCAN algorithm with optimal parameters. The immune cell density, cell-to-cell contacts, and functional marker intensity in the nest area are performed.

##
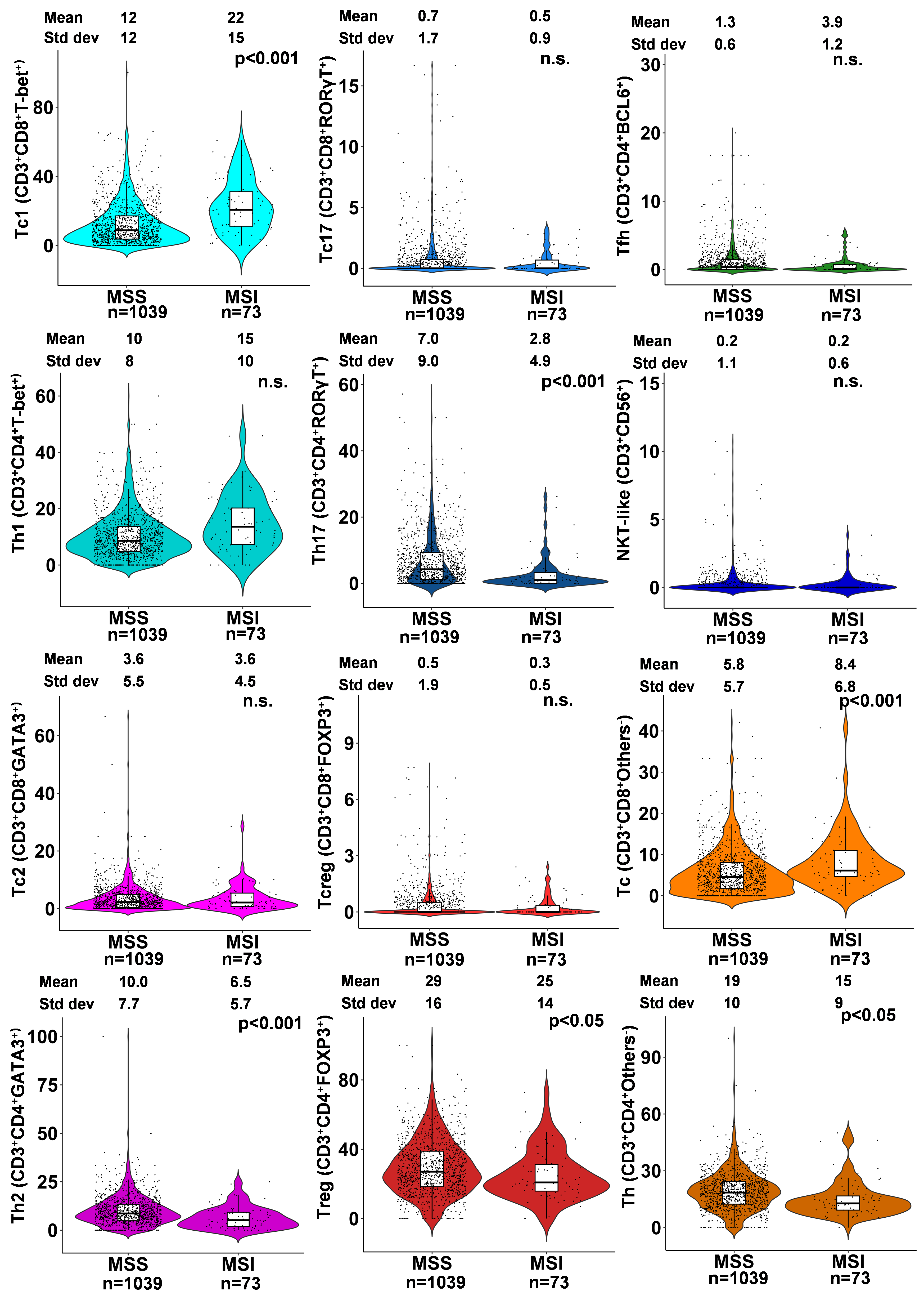


## Figure S3. Fraction of T-cell subsets between MSS and MSI colorectal cancers.

## The fraction (%) of T-cell subsets is shown between MSS and MSI colorectal cancers. Error bars: SEM of each fraction.

##

## Figure S4. Representative images for MSI and MSS cores.


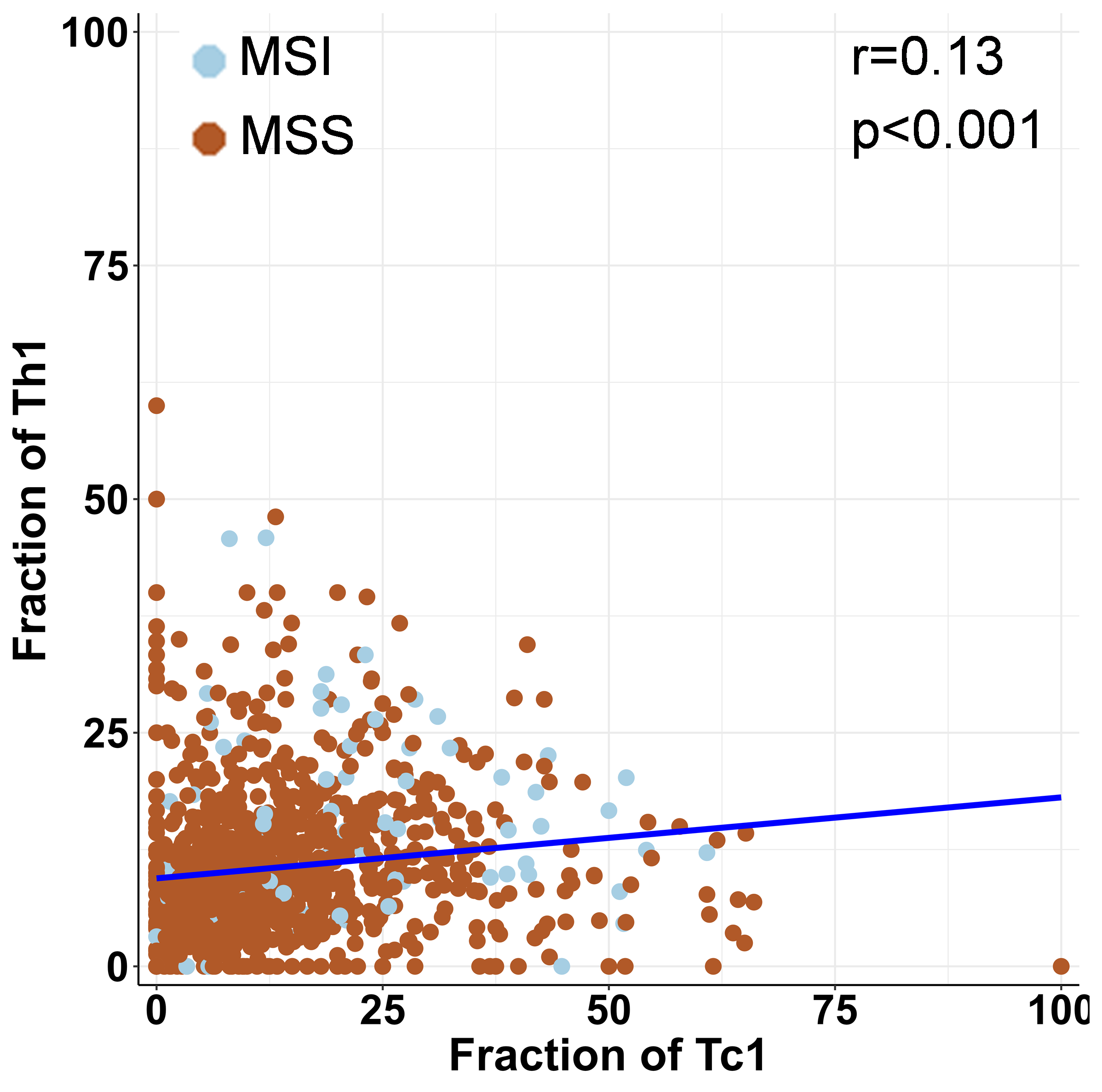


## Figure S5. Association between fraction of Tc1 and Th1.

## Correlation plot showing association between fraction of Tc1 and Th1 in overall patients with MSI and MSS groups.

##
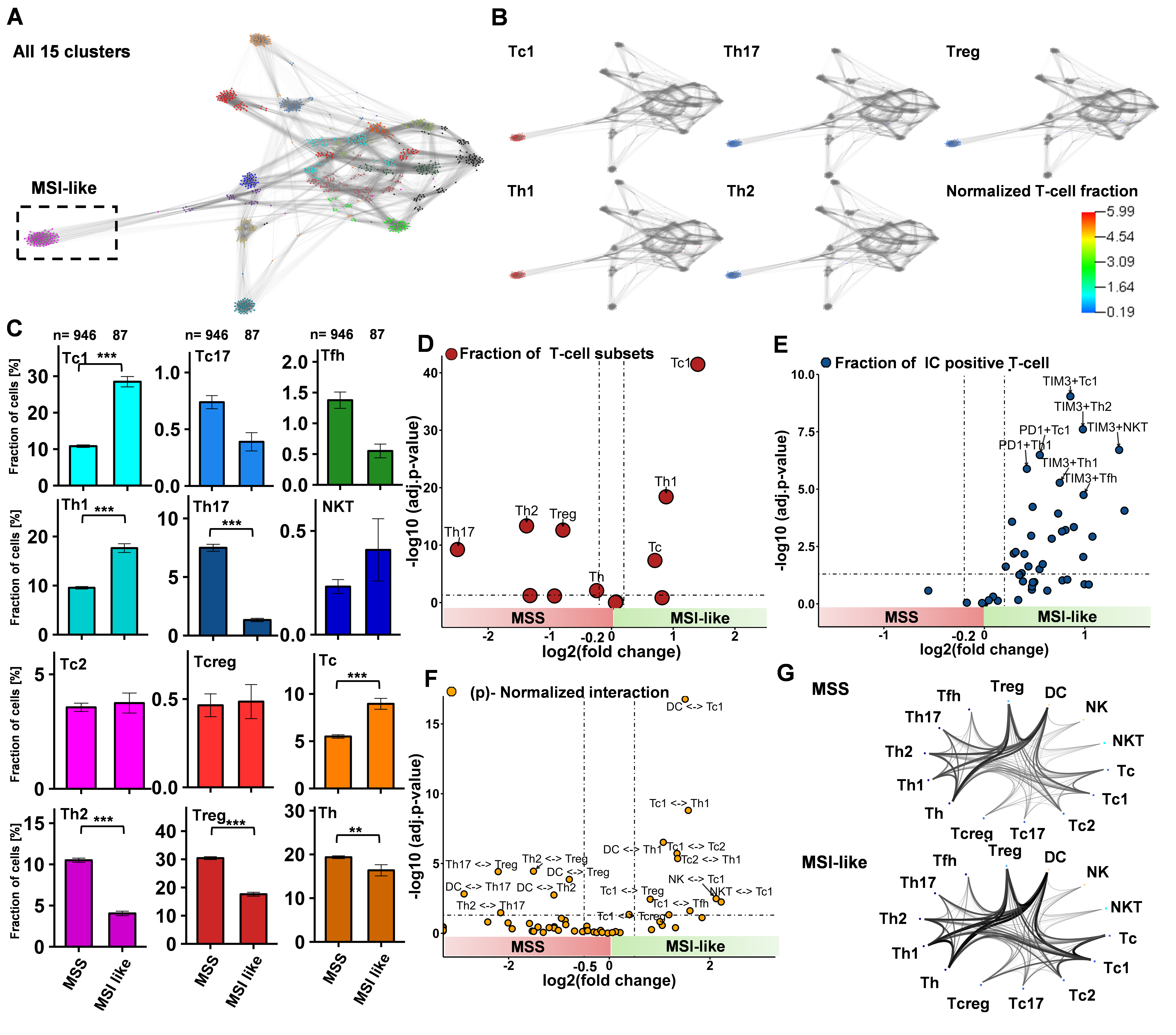


## Figure S6. MSI-like immune phenotype in subcohort of MSS patients.

## (A and B) X-shift clustering of MSS patients identified 87 (8.4%) patients with MSI-like T-cell composition.

## (C) The fraction (%) of T-cell subsets is shown between MSS and MSI-like within MSS colorectal cancers. Error bars: SEM of each proportion. **p* < 0.05, ***p* < 0.01, and ****p* < 0.001.

## (D–F) Volcano plots depicting log^2^ fold-change on *x*-axis and −log^10^ adjusted *p* values on *y*-axis of T-cell fractions (D), functional marker expression (E), and (p)-normalized cell-to-cell interactions (F) between MSI-like and MSS patients.

## (G) Profile of normalized cell-to-cell interactions of T-cell subsets, dendritic cells, and NK cells in MSI-like patients versus MSS patients.

##
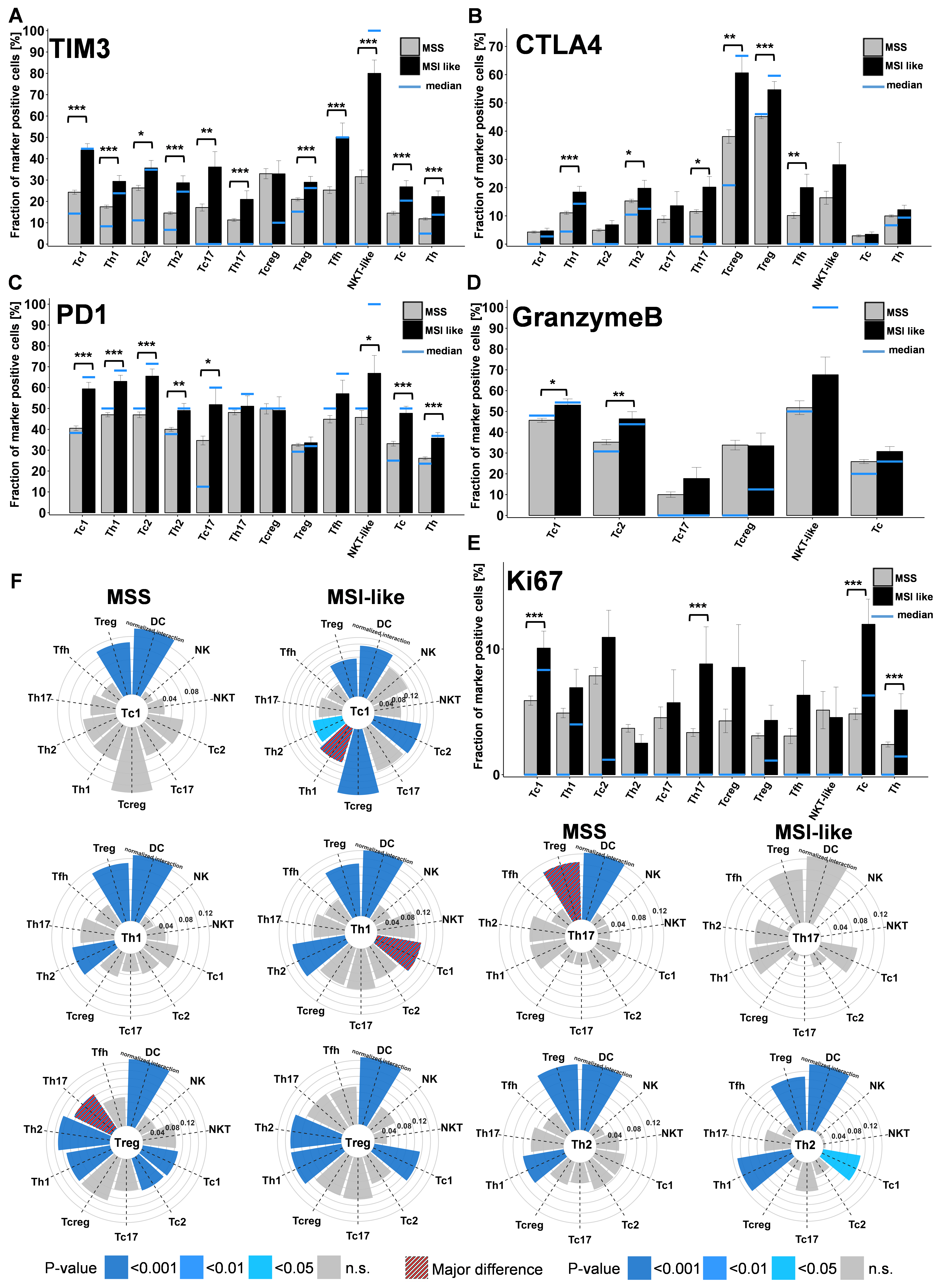


## Figure S7. Functional markers and cell-to-cell interactions between MSI-like and MSS patients.

(A–E) Fraction (%) of marker positive cells is shown for each T-cell subpopulation between MSI-like (black) and MSS (gray) patients. The blue segments inside or outside the bars indicate the median value of each fraction. Error bars: SEM of each fraction. **p* < 0.05, ***p* < 0.01, and ****p* < 0.001.

(F) Circular bar plots: significance of normalized cell-to-cell interactions compared to background noise (size, shades of blue and gray), and significant differences between MSS and MSI-like are highlighted in red.

##
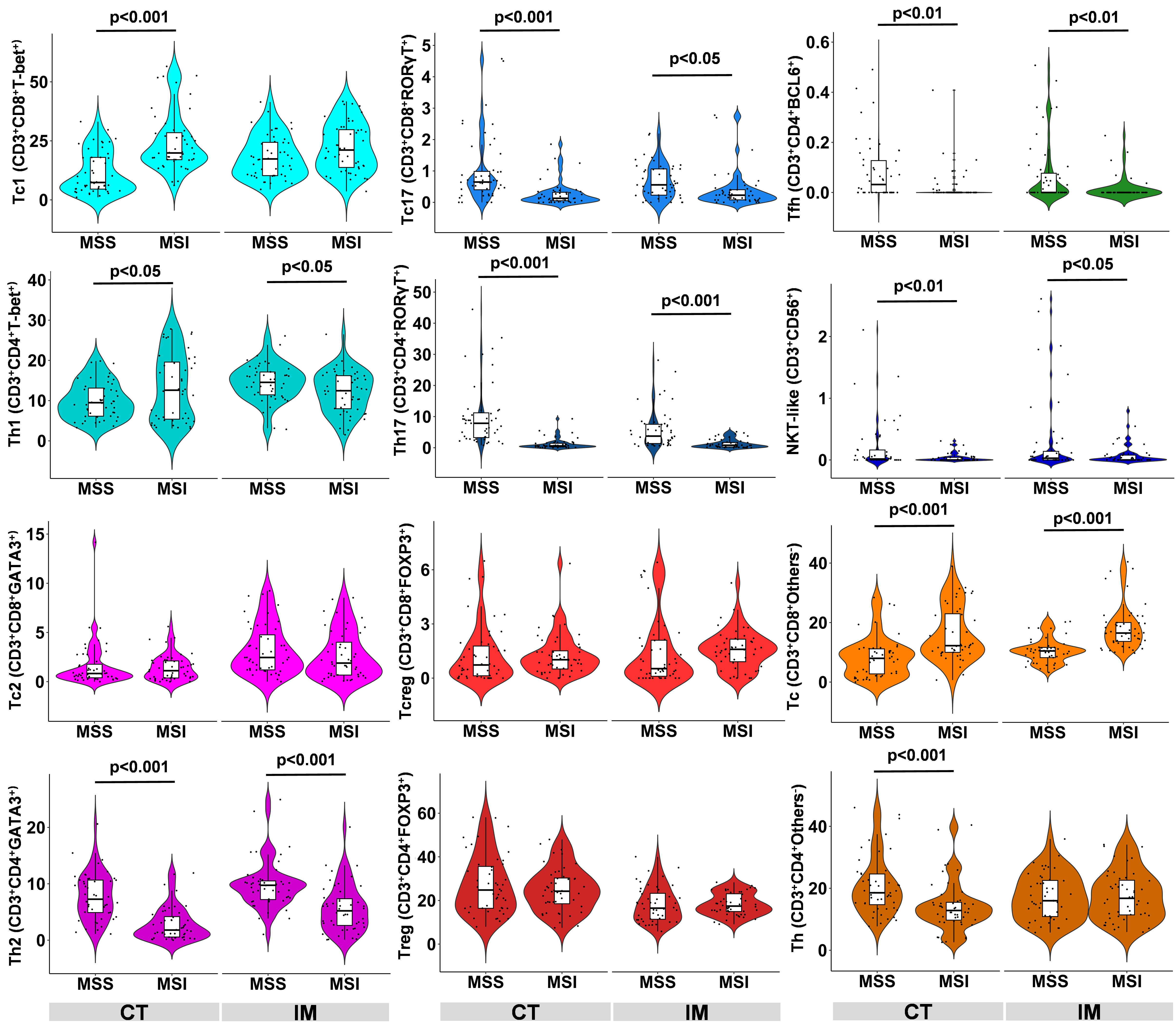


## Figure S8. Difference of fraction of T-cell subsets between MSI and MSS patients in CT versus IM.

The fraction (%) of T-cell subsets is shown between MSS and MSI colorectal cancers in both the center of the tumor (CT) and at the invasive margin (IM) across 12 large sections. Error bars: SEM of each fraction.


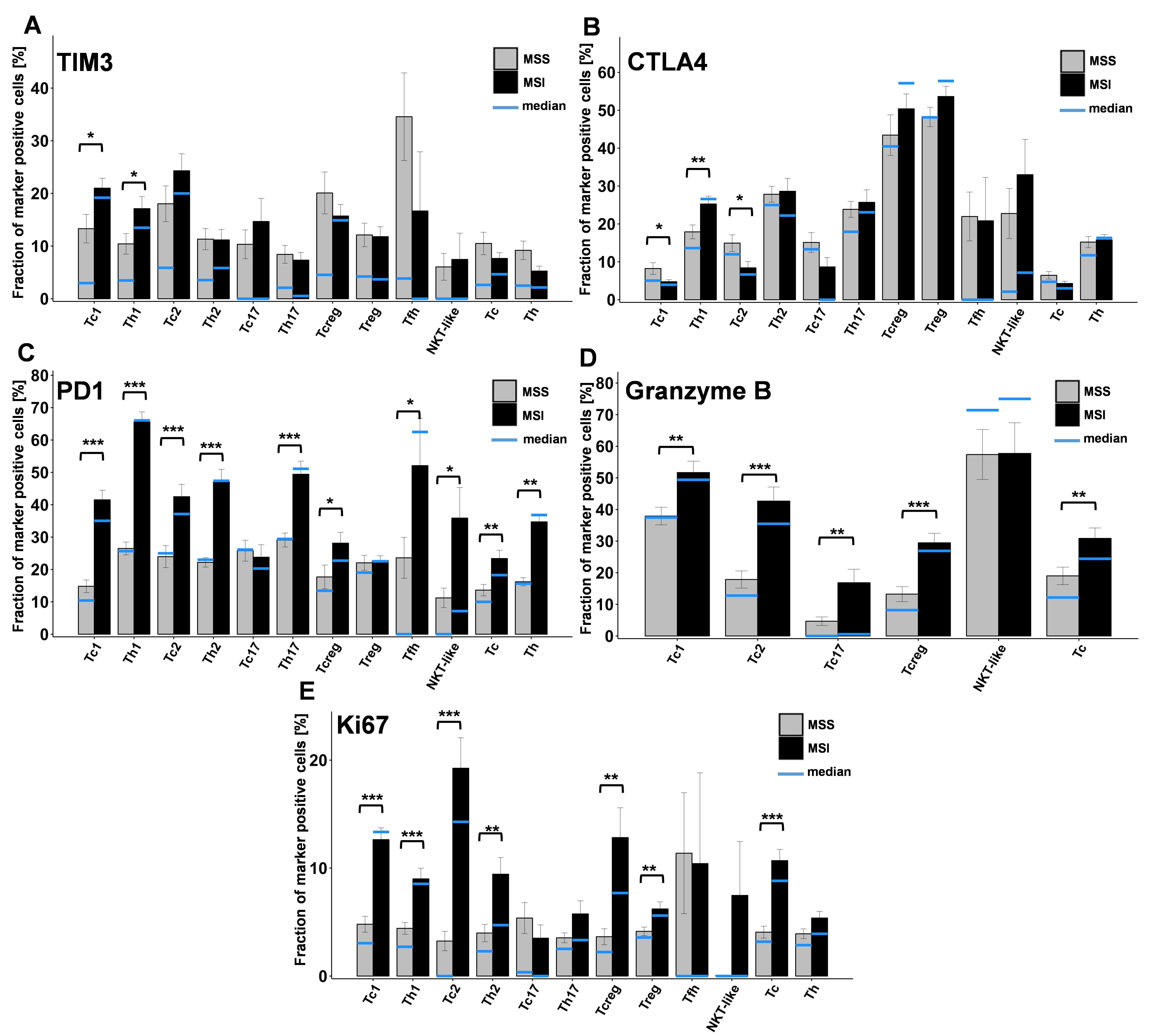


## Figure S9. Functional markers between MSI and MSS patients in center of tumor of large sections.

(A–E) Fraction (%) of marker positive cells is shown for each T-cell subset between MSI (black) and MSS (gray) patients. The blue segments inside or outside the bars indicate the median value of each fraction. Error bars: SEM of each fraction. **p* < 0.05, ***p* < 0.01, and ****p* < 0.001.

## Supplementary Tables S1–S4

## Table S1. Patient characteristics shown for 0.6-mm TMA cores analyzed.

|  | No. patients (%) | |
| --- | --- | --- |
| **Patient characteristics** | **Total study cohort on TMA (*n* = 1,297)** | |
| Sex |  |  |
| Male | 351 | (27.1%) |
| Female | 264 | (20.4%) |
| Missing data | 682 | (52.5%) |
| Microsatellite status – No. (%) |  |  |
| Microsatellite stable (MSS) | 1,203 | (92.8%) |
| Microsatellite instable (MSI) | 94 | (7.2%) |
| Missing data | 0 | (0%) |
| pT stage – No. (%) |  |  |
| pT1 | 53 | (4.1%) |
| pT2 | 268 | (20.7%) |
| pT3 | 708 | (54.6%) |
| pT4 | 257 | (19.8%) |
| Missing data | 11 | (0.8%) |
| pN stage – No. (%) |  |  |
| pN- | 669 | (51.6%) |
| pN+ | 605 | (46.6%) |
| Missing data | 23 | (1.8%) |
| V stage – No. (%) |  |  |
| V0 | 930 | (71.7%) |
| V+ | 335 | (25.8%) |
| Missing data | 32 | (2.5%) |
| L stage – No. (%) |  |  |
| L0 | 525 | (40.5%) |
| L1 | 723 | (55.7%) |
| Missing data | 49 | (3.8%) |
| RAS mutation – No. (%) |  |  |
| Yes | 309 | (23.8%) |
| No | 401 | (30.9%) |
| Missing data | 587 | (45.3%) |
| HER2 amplification – No. (%) |  |  |
| Positive | 17 | (1.3%) |
| Negative | 618 | (47.6%) |
| Missing data | 662 | (51.1%) |

A single 0.6 mm core represents a colorectal cancer specimen in the cohorts.

## Table S2. List of used antibodies, antigen retrieval (AR), dilutions, and Opal dyes for multiplex fluorescence immunohistochemistry.

| **Antibody target** | **Identifier** | **AR (pH value)** | **Dilution** | **Staining position** | **Opal dye** | **BLEACH&STAIN** |
| --- | --- | --- | --- | --- | --- | --- |
| RoRγT | Millipore, Clone: 6F3.1 Catalogue No.: MABF81 | 7.8 | 1:450 | 1 | 520 | Cycle 1 |
| TIM-3 | MSVA, Clone: MSVA-366R Catalogue No.: SKU: 3484-366R-01 | 7.8 | 1:100 | 2 | 570 | Cycle 1 |
| GATA3 | MSVA, Clone: MSVA-550R Catalogue No.: 3209-550R-01 | 7.8 | 1:75 | 3 | 620 | Cycle 1 |
| T-bet | Epitomics, Clone: EP263 Catalogue No.: AC-0240A | 9.0 | 1:150 | 4 | 690 | Cycle 1 |
| CTLA-4 | MSVA, Clone: MSVA-152R Catalogue No.: SKU: 3451-152R-01 | 9.0 | 1:100 | 5 | 520 | Cycle 2 |
| PD-1 | abcam, Clone: [EPR4877(2)] Catalogue No.: ab137132 | 9.0 | 1:450 | 6 | 570 | Cycle 2 |
| CD56 | MSVA, Clone: MSVA-056R Catalogue No.: SKU: 6173-056R-01 | 9.0 | 1:150 | 7 | 620 | Cycle 2 |
| GranzymB | Leica, Clone: 11F1 Catalogue No.: NCL-L-GRAN-B | 9.0 | 1:50 | 8 | 690 | Cycle 2 |
| CD27 | MSVA, Clone: MSVA-027M Catalogue No.: SKU: 2503-027M-01 | 9.0 | 1:450 | 9 | 520 | Cycle 3 |
| CD3 | DAKO, Clone: CD3 Catalogue No.: IR503 | 9.0 | 1:3 | 10 | 570 | Cycle 3 |
| BCL6 | DAKO, Clone: PG-B6p Catalogue No.: IR625 | 9.0 | RTU | 11 | 620 | Cycle 3 |
| CD11c | abcam, Clone: [EP[347Y] Catalogue No.: ab52632 | 9.0 | 1:1000 | 12 | 690 | Cycle 3 |
| FOXP3 | BioLegend, Clone: 206D Catalogue No.: 320102 | 9.0 | 1:75 | 13 | 520 | Cycle 4 |
| CD4 | MSVA, Clone: MSVA-004R Catalogue No.: SKU: 2278-004R-01 | 9.0 | 1:150 | 14 | 570 | Cycle 4 |
| CD8 | DAKO, Clone: C8/144B Catalogue No.: IR623 | 9.0 | 1:25 | 15 | 690 | Cycle 4 |
| panCK | MSVA, Clone: MSVA-000R Catalogue No.: SKU: 2105-000R-01 | 9.0 | 1:600 | 16 | 520 | Cycle 5 |
| Ki67 | MSVA, Clone: MSVA-267M Catalogue No.: SKU: 2082-267M-01 | 9.0 | 1:150 | 17 | 570 | Cycle 5 |
| HLA-DR | MSVA, Clone: MSVA-470R Catalogue No.: SKU: 3328-470R-01 | 9.0 | 1:150 | 18 | 620 | Cycle 5 |
| CD31 | MSVA, Clone: MSVA-031M Catalogue No.: SKU: 2517-031M-01 | 9.0 | 1:250 | 19 | 690 | Cycle 5 |
| (MSVA: MS Validated Antibodies GmbH, AR: antigen retrieval) | | | | | | |

## Table S3. Association of pT, pN with fractions of T-cell subsets in MSS patients.

| **Parameter** | **No. patients analyzed** | **Tc1 fraction (%)** | | | **Th1 fraction (%)** | | | **Tc2 fraction (%)** | | | **Th2 fraction (%)** | | | **Tc17 fraction (%)** | | | **Th17 fraction (%)** | | |
| --- | --- | --- | --- | --- | --- | --- | --- | --- | --- | --- | --- | --- | --- | --- | --- | --- | --- | --- | --- |
|  |  | **Mean (±SD)** | | ***P* value** | **Mean (±SD)** | | **p-value** | **Mean (±SD)** | | ***P* value** | **Mean (±SD)** | | ***P* value** | **Mean (±SD)** | | ***P* value** | **Mean (±SD)** | | ***P* value** |
|  |  |  |  |  |  |  |  |  |  |  |  |  |  |  |  |  |  |  |  |
| **pT-Stage** |  |  |  | **0.2** |  |  | **0.07** |  |  | **<0.001** |  |  | **0.007** |  |  | **0.01** |  |  | **0.03** |
| pT1 | 38 | 14.32 | (±10.82) |  | 8.63 | (±4.76) |  | 3.98 | (±4.51) |  | 7.75 | (±4.75) |  | 1.59 | (±2.76) |  | 10.00 | (±9.51) |  |
| pT2 | 207 | 11.53 | (±10.98) |  | 9.26 | (±7.24) |  | 2.92 | (±2.95) |  | 9.36 | (±6.63) |  | 0.68 | (±1.58) |  | 7.65 | (±7.86) |  |
| pT3 | 576 | 11.88 | (±11.53) |  | 10.31 | (±8.26) |  | 3.32 | (±4.54) |  | 9.80 | (±7.85) |  | 0.72 | (±1.82) |  | 6.95 | (±9.3) |  |
| pT4 | 204 | 13.52 | (±14.29) |  | 11.18 | (±9.29) |  | 4.91 | (±8.87) |  | 11.45 | (±8.76) |  | 0.57 | (±1.26) |  | 5.84 | (±8.86) |  |
| **pN-Stage** |  |  |  | **0.3** |  |  | **0.3** |  |  | **0.99** |  |  | **0.02** |  |  | **0.62** |  |  | **0.84** |
| pN- | 510 | 12.59 | (±11.81) |  | 10.50 | (±7.87) |  | 3.58 | (±6.19) |  | 9.37 | (±6.35) |  | 0.74 | (±1.81) |  | 6.89 | (±7.64) |  |
| pN+ | 506 | 11.80 | (±12.18) |  | 9.96 | (±8.57) |  | 3.59 | (±4.71) |  | 10.53 | (±8.91) |  | 0.68 | (±1.63) |  | 7.00 | (±10.11) |  |

| **Parameter** | **No. patients analyzed** | **Tcreg fraction (%)** | | | **Treg fraction (%)** | | | | **Tfh fraction (%)** | | | | | **NKT-like fraction (%)** | | | | | **Tc fraction (%)** | | | | | **Th fraction (%)** | | | | | |
| --- | --- | --- | --- | --- | --- | --- | --- | --- | --- | --- | --- | --- | --- | --- | --- | --- | --- | --- | --- | --- | --- | --- | --- | --- | --- | --- | --- | --- | --- |
|  |  | **Mean (±SD)** | | ***P* value** | **Mean (±SD)** | | ***P* value** | | **Mean (±SD)** | | | ***P* value** | | **Mean (±SD)** | | | ***P* value** | | **Mean (±SD)** | | | ***P* value** | | **Mean (±SD)** | | | ***P* value** | |  |
|  |  |  |  |  |  |  |  |  |  |  |  |  |  |  |  |  |  |  |  |  |  |  |  |  |  |  |  |  |  |
| **pT-Stage** |  |  |  | **0.12** |  |  | **<**  **0.001** | |  |  | | **0.11** | |  |  | | **0.64** | |  |  | | **0.17** | |  |  | | **0.06** | |  |
| pT1 | 38 | 0.56 | (±0.76) |  | 26.57 | (±11.35) |  |  | 1.39 | (±2.27) | |  |  | 0.25 | (±0.38) | |  |  | 5.41 | (±3.56) | |  |  | 19.54 | (±6.38) | |  |  |  |
| pT2 | 207 | 0.52 | (±1.6) |  | 29.59 | (±14.05) |  |  | 1.04 | (±1.82) | |  |  | 0.32 | (±1.08) | |  |  | 6.46 | (±6.29) | |  |  | 20.68 | (±9.99) | |  |  |  |
| pT3 | 576 | 0.35 | (±0.91) |  | 30.87 | (±16.36) |  |  | 1.19 | (±2.57) | |  |  | 0.21 | (±0.83) | |  |  | 5.49 | (±5.3) | |  |  | 18.90 | (±9.69) | |  |  |  |
| pT4 | 204 | 0.73 | (±3.69) |  | 25.43 | (±16.1) |  |  | 1.90 | (±7.4) | |  |  | 0.28 | (±1.65) | |  |  | 6.07 | (±6.39) | |  |  | 18.13 | (±11.74) | |  |  |  |
| **pN-Stage** |  |  |  | **0.95** |  |  | **0.51** | |  |  | | **0.03** | |  |  | | **0.96** | |  |  | | **0.25** | |  |  | | **0.27** | |  |
| pN- | 510 | 0.47 | (±1.25) |  | 29.05 | (±15.1) |  |  | 1.04 | (±1.95) | |  |  | 0.25 | (±0.87) | |  |  | 6.03 | (±5.61) | |  |  | 19.51 | (±9.28) | |  |  |  |
| pN+ | 506 | 0.48 | (±2.44) |  | 29.71 | (±16.59) |  |  | 1.59 | (±5.24) | |  |  | 0.25 | (±1.26) | |  |  | 5.61 | (±5.8) | |  |  | 18.80 | (±10.91) | |  |  |  |
| **p* ≤ 0.05, ***p* ≤ 0.01, ****p* ≤ 0.001. | | |  |  |  |  | |  |  | |  | |  |  | |  | |  |  | |  | |  |  | |  | |  | |

## Table S4. Association of pT, pN with fractions of T-cell subsets in MSI patients.

| **Parameter** | **No. patients analyzed** | **Tc1 fraction (%)** | | | **Th1 fraction (%)** | | | **Tc2 fraction (%)** | | | **Th2 fraction (%)** | | | **Tc17 fraction (%)** | | | **Th17 fraction (%)** | | |
| --- | --- | --- | --- | --- | --- | --- | --- | --- | --- | --- | --- | --- | --- | --- | --- | --- | --- | --- | --- |
|  |  | **Mean (±SD)** | | ***P* value** | **Mean (±SD)** | | ***P* value** | **Mean (±SD)** | | ***P* value** | **Mean (±SD)** | | ***P* value** | **Mean (±SD)** | | ***P* value** | **Mean (±SD)** | | ***P* value** |
|  |  |  |  |  |  |  |  |  |  |  |  |  |  |  |  |  |  |  |  |
| **pT-Stage** |  |  |  | **0.81** |  |  | **0.2** |  |  | **0.84** |  |  | **0.51** |  |  | **0.47** |  |  | **0.59** |
| pT1 | 6 | 19.69 | (±12.86) |  | 15.61 | (±7.63) |  | 4.41 | (±3.69) |  | 7.42 | (±2.74) |  | 0.81 | (±1.11) |  | 4.31 | (±4.28) |  |
| pT2 | 16 | 22.55 | (±17.51) |  | 11.89 | (±8.01) |  | 2.78 | (±3.07) |  | 6.93 | (±5.37) |  | 0.46 | (±0.77) |  | 3.52 | (±5.94) |  |
| pT3 | 36 | 21.37 | (±15.29) |  | 14.22 | (±10.81) |  | 3.72 | (±5.33) |  | 6.92 | (±6.9) |  | 0.58 | (±0.99) |  | 2.68 | (±5.27) |  |
| pT4 | 15 | 25.52 | (±13.6) |  | 19.49 | (±10.56) |  | 3.97 | (±3.9) |  | 4.46 | (±3.01) |  | 0.22 | (±0.52) |  | 1.51 | (±2.6) |  |
| **pN-Stage** |  |  |  | **0.99** |  |  | **0.03** |  |  | **0.003** |  |  | **0.66** |  |  | **0.6** |  |  | **0.76** |
| pN- | 46 | 22.00 | (±14.33) |  | 17.00 | (±9.89) |  | 2.36 | (±2.89) |  | 6.25 | (±4.9) |  | 0.50 | (±0.87) |  | 2.89 | (±4.76) |  |
| pN+ | 25 | 22.06 | (±16.85) |  | 11.47 | (±10.06) |  | 5.53 | (±5.92) |  | 6.89 | (±7.16) |  | 0.39 | (±0.8) |  | 2.50 | (±5.44) |  |

| **Parameter** | **No. patients analyzed** | **Tcreg fraction (%)** | | | | **Treg fraction (%)** | | | | | | **Tfh fraction (%)** | | | | | | **NKT-like fraction (%)** | | | | | | **Tc fraction (%)** | | | | | | **Th fraction (%)** | | | | | |
| --- | --- | --- | --- | --- | --- | --- | --- | --- | --- | --- | --- | --- | --- | --- | --- | --- | --- | --- | --- | --- | --- | --- | --- | --- | --- | --- | --- | --- | --- | --- | --- | --- | --- | --- | --- |
|  |  | **Mean (±SD)** | | ***P* value** | | **Mean (±SD)** | | | | ***P* value** | | **Mean (±SD)** | | | | ***P* value** | | **Mean (±SD)** | | | | ***P* value** | | **Mean (±SD)** | | | | ***P* value** | | **Mean (±SD)** | | | | ***P* value** | |
|  |  |  |  |  |  |  |  |  |  |  |  |  |  |  |  |  |  |  |  |  |  |  |  |  |  |  |  |  |  |  |  |  |  |  |  |
| **pT-Stage** |  |  |  | **0.01** | |  | |  | | **0.74** | |  | |  | | **0.42** | |  | |  | | **0.19** | |  | |  | | **0.9** | |  | |  | | **0.91** | |
| pT1 | 6 | 0.29 | (±0.29) |  |  | 22.49 | | (±9.51) | |  |  | 0.39 | | (±0.3) | |  |  | 0.02 | | (±0.04) | |  |  | 7.93 | | (±3.03) | |  |  | 16.64 | | (±7.4) | |  |  |
| pT2 | 16 | 0.36 | (±0.45) |  |  | 27.41 | | (±9.76) | |  |  | 0.59 | | (±1.25) | |  |  | 0.14 | | (±0.3) | |  |  | 7.54 | | (±4.24) | |  |  | 15.81 | | (±10.44) | |  |  |
| pT3 | 36 | 0.11 | (±0.34) |  |  | 25.47 | | (±17.15) | |  |  | 0.84 | | (±1.44) | |  |  | 0.39 | | (±0.87) | |  |  | 9.00 | | (±8.44) | |  |  | 14.71 | | (±10.54) | |  |  |
| pT4 | 15 | 0.63 | (±0.82) |  |  | 22.10 | | (±12.74) | |  |  | 0.25 | | (±0.45) | |  |  | 0.01 | | (±0.05) | |  |  | 8.00 | | (±5.94) | |  |  | 13.84 | | (±6.1) | |  |  |
| **pN-Stage** |  |  |  | **0.92** | |  | |  | | **0.22** | |  | |  | | **0.77** | |  | |  | | **0.23** | |  | |  | | **0.78** | |  | |  | | **0.23** | |
| pN- | 46 | 0.29 | (±0.54) |  |  | 23.66 | | (±13.03) | |  |  | 0.66 | | (±1.16) | |  |  | 0.16 | | (±0.43) | |  |  | 8.14 | | (±6.87) | |  |  | 16.07 | | (±9.35) | |  |  |
| pN+ | 25 | 0.28 | (±0.52) |  |  | 28.11 | | (±16.53) | |  |  | 0.58 | | (±1.33) | |  |  | 0.36 | | (±0.92) | |  |  | 8.63 | | (±7.04) | |  |  | 13.21 | | (±9.62) | |  |  |
| **p* ≤ 0.05, ***p* ≤ 0.01, ****p* ≤ 0.001. | | | | |  | |  | |  | |  | |  | |  | |  | |  | |  | |  | |  | |  | |  |  |  | |  | |  |
